# Ethanol as a Modifier of Drug Toxicity in Humans: Pathways of Toxicity and Organ-Level Consequences

**DOI:** 10.3390/ijms27146270

**Published:** 2026-07-14

**Authors:** Bożena Bukowska, Karol Bukowski, Marlena Broncel

**Affiliations:** 1 Department of Biophysics of Environmental Pollution, Faculty of Biology and Environmental Protection, University of Lodz, ul. Pomorska 141/143, 90–236 Lodz, Poland; 2 Department of Medical Biophysics, Faculty of Biology and Environmental Protection, University of Lodz, ul. Pomorska 141/143, 90–236 Lodz, Poland; karol.bukowski@biol.uni.lodz.pl; 3 Department of Internal Diseases and Clinical Pharmacology, Medical University of Lodz, Kniaziewicza 1/5, 91–347 Lodz, Poland; marlena.broncel@umed.lodz.pl

**Keywords:** alcohol, CES1, clinical pharmacology, CYP2E1, drug metabolism, ethanol–drug interactions

## Abstract

Ethanol consumption can modify both drug exposure and drug response. However, the clinical relevance of these interactions depends strongly on the timing and pattern of alcohol intake, the affected pharmacological pathway, the dosage form and organ reserve. This review summarizes current evidence on ethanol–drug interactions, particularly human crossover studies, phenotyping studies, cohort analyses and appropriate case reports. It distinguishes acute ethanol–drug co-exposure, chronic alcohol exposure, drug use during early abstinence after chronic drinking, and pharmacotherapy in alcohol-associated liver disease. Key mechanisms include ADH- and ALDH-dependent ethanol oxidation, acetaldehyde formation, NADH/NAD^+^ redox shift, CYP2E1 induction, carboxylesterase 1 (CES1) modulation, altered intestinal and hepatic first-pass handling, dose dumping from susceptible modified-release products, changes in protein binding in alcohol-associated liver disease, and ALDH inhibition with acetaldehyde accumulation in disulfiram-like reactions. At the molecular level, ethanol may promote acetaldehyde adduct formation with proteins and DNA, CYP2E1-driven reactive oxygen species generation, redox stress, intestinal barrier injury, and CES1-dependent transesterification of selected ester drugs. Acute ethanol intake mainly increases pharmacodynamic toxicity and causes short-term pharmacokinetic disturbances, including enhanced central nervous system depression, delayed gastric emptying, impaired glucose and lactate handling and altered hemodynamic responses. In contrast, chronic exposure, early abstinence and alcohol-associated liver disease are more often associated with hepatic enzyme and transporter remodeling, altered protein binding, reduced hepatic or renal reserve, and greater susceptibility to drug-related organ injury. The highest-risk scenarios involve older adults, polypharmacy, alcohol-associated liver disease, dehydration or acute illness, early abstinence, and the concurrent use of central nervous system depressants, glucose-lowering drugs, NSAIDs, antihypertensives, renally eliminated drugs or warfarin. Hence, ethanol exposure should be treated as a dynamic, context-dependent modifier factor that can acutely exacerbate pharmacodynamic toxicity, alter selected pharmacokinetic pathways and lower organ tolerance to drug-related injury.

## 1. Introduction

Alcohol use remains a major public health problem. In 2019, alcohol consumption was responsible for approximately 2.6 million deaths worldwide, corresponding to 4.7% of all deaths, and approximately 209 million people aged 15 years and older were estimated to live with alcohol dependence [1]. Alcohol-related harm disproportionately affects younger adults and remains a major contributor to the burden attributable to alcohol and mortality in Europe, particularly in Eastern Europe [2,3]. This is closely associated with drug safety, as alcohol consumption may coincide with drug co-exposure in patients using prescription, over-the-counter, and centrally acting medications. In Poland, recent national reports indicate annual alcohol consumption of around 10 L of pure alcohol per capita and highlight continuing concern about alcohol-related mortality and the affordability and availability of alcohol [4]. Ethanol is rapidly absorbed and distributed and is eliminated mainly through ADH- and ALDH-dependent oxidation; its metabolism also varies with regard to drinking pattern, nutritional status, sex, body composition, hepatic metabolic capacity and ADH/ALDH genetic variation. These factors may modify acetaldehyde exposure, functional impairment and susceptibility to ethanol–drug interactions. As such, ethanol consumption should be considered a systemically distributed, psychoactive and metabolically active agent that can cause co-exposure during pharmacotherapy [5,6,7].

Accordingly, blood alcohol concentration and the rate of ethanol elimination should be interpreted as dynamic exposure markers that do not fully capture functional impairment, pharmacological vulnerability, or interaction risk. Thus, alcohol–medication interactions are better understood as context-dependent pharmacological events rather than as generic “avoid alcohol” warnings.

As alcohol consumption is often associated with multimorbidity, chronic pharmacotherapy, and polypharmacy, especially in older adults, the simple statement that a patient “drinks” is insufficient for risk assessment. Alcohol consumption can modify drug safety through pharmacodynamic additivity and altered drug absorption and metabolism, with the risk further modified by organ dysfunction, dehydration, malnutrition, liver disease and polypharmacy [8,9,10,11]. These risks are particularly relevant in older adults, as reflected in the POSAMINO framework and subsequent studies [9,12,13]. Ethanol consumption can have a number of effects on concurrent pharmacotherapy. Ethanol itself acts on the central nervous system, is rapidly distributed, undergoes oxidative metabolism and alters the cellular redox balance [14,15]; it can also alter cytochrome P450 activity [16,17,18,19,20] and inhibit selected carboxylesterases [21]. Patients with advanced alcohol-associated liver disease may also have impaired synthetic function and hypoalbuminemia which can further modify drug distribution and exposure [22]. Ethanol may also increase susceptibility to certain drug-related liver and kidney injury. However, this risk is context-dependent and can vary considerably between timing-dependent paracetamol/acetaminophen hepatotoxicity, DILI occurring in the context of alcohol exposure or alcohol-associated liver disease and hemodynamic AKI involving dehydration, NSAIDs, RAS inhibitors or diuretics [23,24,25,26,27,28].

This review summarizes the current understanding of the toxicology and clinical pharmacology of ethanol–drug interactions. It treats acute ethanol exposure, chronic heavy use, abstinence after chronic exposure, and alcohol-associated liver disease as distinct clinical states that exert different, and sometimes opposing, effects on drug exposure and toxicity. The paper focuses on the mechanisms by which ethanol modifies drug exposure, pharmacodynamic response, toxicity and organ vulnerability; these include ADH/ALDH-dependent oxidation, acetaldehyde formation, NADH/NAD^+^ redox shift, CYP2E1 induction, CES1 modulation, altered first-pass handling, formulation-dependent dose dumping, protein binding changes and reduced organ reserve.

## 2. Search Strategy and Evidence Appraisal

This article is intended as a narrative review focusing on the biological, toxicological and clinical–pharmacological basis of ethanol–drug interactions rather than as a systematic review or meta-analysis; as such, it did not follow PRISMA guidelines, nor did it include any formal risk-of-bias assessment or quantitative synthesis. To improve transparency, the review corpus was identified mainly through PubMed/MEDLINE and by citation tracking of key articles. Regulatory documents, prescribing information and product-specific safety reports were also considered when they were relevant to formulation-dependent interactions or clinical safety. The literature searches covered publications available up to May 2026. Publications from 2000 to May 2026 were prioritized, but older landmark pharmacokinetic, pharmacodynamic and regulatory studies were retained when they remained important for current interpretation. The following search terms were used: ethanol, alcohol, drug interaction, pharmacokinetic, pharmacodynamic, CYP2E1, CYP3A, CYP1A2, CYP2D6, carboxylesterase, CES1, dose dumping, modified-release formulation, disulfiram-like reaction, alcohol-associated liver disease, cirrhosis, drug-induced liver injury, acute kidney injury, gastrointestinal bleeding, metformin, SGLT2 inhibitors, warfarin, paracetamol, acetaminophen, methylphenidate, oseltamivir, benzodiazepines, opioids, NSAIDs, older adults and polypharmacy.

Studies were included when they addressed ethanol-related changes in drug exposure, drug response, metabolic pathway activity, organ vulnerability or toxicity relevant to human health. Priority was given to the following articles: randomized crossover trials, controlled pharmacokinetic/pharmacodynamic studies, ethanol-challenge studies, formulation-interaction studies, human phenotyping studies, cohort and case–control studies, systematic reviews and meta-analyses, translational studies using human tissues or relevant biological models, or studies based on human tissues or relevant pathways. Case reports and short case series were included only when they provided a clear temporal relationship with ethanol exposure, sufficient clinical detail and biological plausibility.

Evidence strength was assessed qualitatively according to study design. For consistency across the tables, studies were classified as controlled human evidence, observational clinical evidence, case-report evidence, or supporting biological evidence. Controlled human studies were treated as the strongest evidence for direct ethanol–drug interactions. Observational studies were used to identify associations relevant to patient care, but not regarded as proof of causality. Case reports were treated as clinical signals rather than as causal evidence. Biological studies were used mainly to support biological plausibility.

## 3. Exposure-Pattern Framework for Ethanol–Drug Interactions

Ethanol–drug interactions depend not only on the drug or molecular pathway involved, but also on the pattern, timing and clinical context of alcohol exposure. Therefore, this review distinguishes four scenarios that can influence drug safety in humans: acute ethanol–drug co-exposure, chronic alcohol exposure, early abstinence or withdrawal after chronic drinking, and the use of pharmacotherapy in alcohol-associated liver disease. By using this framework, it is possible to separate direct acute interactions from chronic adaptive and disease-related changes.

The same medication may behave differently when ethanol is present at the time of dosing, when the patient has engaged in repeated heavy drinking or shortly after cessation of chronic alcohol intake; it may also differ in patients with alcohol-associated liver disease [8,16,29,30].

During acute co-exposure, ethanol may act as a pharmacodynamic amplifier, a transient metabolic competitor, or a modifier of oral drug release and absorption [17,21,31,32]. This acute pattern reflects the presence of ethanol at or near the time of drug dosing. It may involve transient competition for metabolic pathways, pharmacodynamic additivity, altered first-pass metabolism, or non-CYP interactions such as CES1-mediated hydrolysis and transesterification. In addition, acute interactions may depend on luminal ethanol concentration and drug formulation, particularly in the case of susceptible modified-release products [31]. This pattern has particular significance for central nervous system depressants, glucose-lowering drugs, vasodilators, modified-release formulations and selected CES1 substrates.

Chronic alcohol exposure is more closely associated with CYP2E1 induction, oxidative stress and broader remodeling of hepatic drug-metabolizing enzymes and transporter-related pathways [16,18,19,20]. Unlike acute co-exposure, chronic alcohol exposure represents an adaptive state in which repeated ethanol intake may modify enzyme abundance, redox balance, inflammatory signaling and hepatic drug-handling capacity.

Early abstinence represents a clinically important transitional state. Although ethanol is no longer present, some metabolic consequences of chronic alcohol use may persist, including CYP2E1 induction, altered redox balance, poor nutritional status and reduced glutathione reserve. Therefore, early abstinence should be regarded as a distinct state, neither equivalent to active ethanol co-exposure nor to complete metabolic recovery [16,18,20,33,34].

Alcohol-associated liver disease may further modify drug disposition through hypoalbuminemia, hyperbilirubinemia, impaired hepatic perfusion, portosystemic shunting, reduced metabolic capacity and lower hepatic or renal functional reserve, especially in advanced disease or cirrhosis [30,35,36,37]. This state should be distinguished from solely chronic alcohol exposure, because cirrhosis and advanced liver disease may have a considerable influence on drug disposition independently of direct ethanol effects. Other interactions reflect acute changes in first-pass metabolism, redox state or pharmacodynamic response. In addition, chronic exposure and alcohol-associated liver disease may alter hepatic enzyme expression, transporter abundance, protein binding and functional clearance [16,18,37,38].

**Figure 1:**
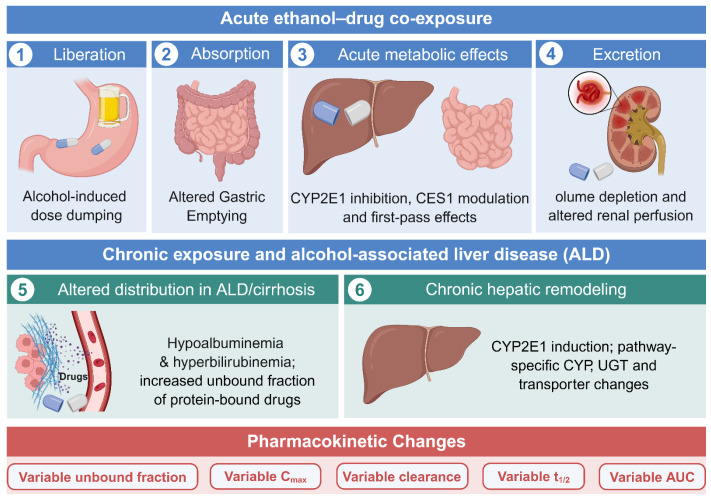
Complementary LADME-based overview of selected ethanol-related effects on drug exposure and pharmacokinetic processes according to exposure pattern. Acute ethanol–drug co-exposure may affect the liberation, absorption, acute metabolic effects and excretion of the drug, whereas chronic exposure and alcohol-associated liver disease may modify its distribution and hepatic drug-handling capacity. The figure is intended as a schematic overview and does not separately depict early abstinence, which is discussed in the text. Created with BioRender.com. Bukowska, B. https://BioRender.com/1sdc0p2 (accessed on 5 July 2026).

These exposure patterns provide the conceptual framework for the following sections. Their main effects across the LADME sequence are summarized in Figure 1.

## 4. Adverse Effects of Acute Ethanol–Drug Co-Exposure

### 4.1. Formulation-Dependent Release and Gastrointestinal Absorption

During acute co-exposure, ethanol may alter oral drug delivery by exerting a direct effect on the dosage form and influencing gastrointestinal physiology. Alcohol does not need to change metabolism to produce toxicity. In susceptible products, ethanol may directly alter the release characteristics of the dosage form. The best-known example is alcohol-induced dose dumping from modified-release formulations. This is not a uniform class effect of all modified-release products. Not every prolonged-release product behaves this way. However, when a formulation is vulnerable, the intended slow-release profile can collapse into an early peak, leading to earlier, and sometimes substantially higher, peak concentrations than the product was intended to produce. D’Souza et al. emphasized that this matters most for drugs with a narrow therapeutic window and for CNS-active agents, especially opioids [31]. Hydromorphone extended-release capsules (Palladone) were withdrawn from the U.S. market after the FDA identified alcohol-induced dose dumping as a serious safety risk [39,40]. Subsequent formulation-specific studies indicate that susceptibility to alcohol-induced dose dumping varies between modified-release products (Table 1).

The controlled studies summarized in Table 1 show that ethanol may alter drug release and/or increase early or peak exposure for some modified-release formulations but not others. Findings from one formulation should therefore not be extrapolated to all other products. Alcohol can also exert its influence on a drug before the tablet or capsule leaves the stomach. In an ultrasonographic study, ethanol-containing beverages, especially red wine, slowed gastric emptying of a solid meal [45]. Delayed gastric emptying may shift the timing of absorption and make peak concentrations less predictable. This may delay delivery of an orally administered drug to the small intestine and shift the time to peak concentration without necessarily increasing total systemic exposure. Thus, acute ethanol can affect oral drug delivery through two mechanistically distinct processes: altered release from a susceptible formulation and delayed gastric transit. However, formulation-specific human evidence is required to determine the final pharmacokinetic effect.

### 4.2. Acute Metabolic Competition and First-Pass Interactions

#### 4.2.1. Pathway-Selective CYP Inhibition During Acute Ethanol Exposure

According to controlled phenotyping studies, acute ethanol exposure does not globally suppress drug metabolism but rather has selective effects. In a randomized crossover probe-drug cocktail study, 16 healthy volunteers received the probe cocktail with repeated co-administration of either vodka or water to achieve an initial blood alcohol concentration of about 0.7 g/L. Ethanol increased dextromethorphan AUC 1.95-fold, increased caffeine AUC 1.38-fold, and reduced intestinal first-pass extraction of midazolam to 77% of the control value, whereas the probe-substrate indices used to assess CYP2C9, CYP2C19, *N*-acetyltransferase 2 (NAT2), and P-glycoprotein (P-gp) activity showed little change [17]. These findings indicate that acute ethanol elicits pathway-selective effects involving CYP1A2, CYP2D6 and intestinal CYP3A rather than causing generalized inhibition of drug metabolism. Because midazolam undergoes both intestinal and hepatic CYP3A-mediated first-pass metabolism, the combined use of oral and intravenous midazolam probe data allows the intestinal component to be estimated; however, this finding should be interpreted as reduced intestinal extraction rather than generalized CYP3A inhibition. Chlorzoxazone phenotyping studies likewise show that acute alcohol exposure alters phenotypic readouts associated with CYP2E1; in particular, acute ethanol reduced the 6-hydroxychlorzoxazone/chlorzoxazone ratio, which is used as an in vivo measure of CYP2E1 activity. This change is consistent with competitive inhibition rather than enzyme induction [46]. A study of 10 healthy volunteers found that acute alcohol intake increased nifedipine AUC by 54% and shortened the time to peak heart-rate response from 2.2 to 1.4 h, indicating that acute ethanol may alter nifedipine exposure and accelerate its hemodynamic effects, potentially through reduced pre-systemic metabolism [47]. As nifedipine undergoes extensive CYP3A-dependent first-pass metabolism, these findings indicate altered pre-systemic handling. However, the study did not directly distinguish intestinal from hepatic CYP3A involvement, and the observed effect should therefore not be attributed exclusively to intestinal CYP3A inhibition.

#### 4.2.2. CES1-Mediated Hydrolysis and Transesterification

During acute co-exposure, ethanol can interfere with CES1-mediated metabolism by inhibiting hydrolysis and, for selected ester drugs, by promoting transesterification. This is supported by human evidence from studies on oseltamivir and methylphenidate, whereas aspirin pharmacokinetics were not meaningfully affected, supporting pathway and substrate selectivity [21,32]. This is consistent with the broader pharmacology of CES1, a major hepatic hydrolase for many ester-containing drugs whose activity varies with substrate specificity, inhibition and genetic variation [48,49,50,51]. Accordingly, ethanol-related CES1 interactions should not be interpreted as a uniform class effect, but as substrate-dependent interactions in which ethanol may reduce CES1-mediated hydrolysis, thereby altering prodrug activation, active-drug inactivation, or promoting transesterification.

This is important because ethanol–drug interactions are not limited to cytochrome P450 enzymes; ethanol can also interfere with non-CYP hydrolytic pathways that are involved in the metabolism and safety of ester drugs. The ethanol–methylphenidate interaction is a useful example of acute metabolic competition at the CES1-dependent first-pass level because it combines reduced hydrolysis of the parent drug with the formation of ethylphenidate. In a study of 24 healthy volunteers, Patrick et al. indicate that co-administration of ethanol (0.6 g/kg) with racemic methylphenidate increased early d-methylphenidate concentrations by 44–99% during the absorption phase and increased total d-methylphenidate AUC by 21%. Participants were also more likely to report subjective effects such as feeling “high” and “stimulated” [32].

Acute ethanol may therefore increase early drug exposure, not only by changing first-pass handling but also by redirecting metabolism toward a different product: the same alcohol exposure can simultaneously increase parent-drug availability and generate a new metabolite with potential pharmacological relevance. This pattern is consistent with the CES1-dependent effects of ethanol [21] and supports the broader view that ethanol–drug interactions are not limited to cytochrome P450 pathways. Representative controlled human studies and experimental evidence concerning acute pathway-selective CYP inhibition and CES1-mediated interactions are summarized in Table 2.

### 4.3. Acute Pharmacodynamic Interactions

During acute co-exposure, ethanol may exert convergent pharmacodynamic effects with drugs. These can affect alertness, psychomotor performance, respiratory drive, glucose homeostasis, autonomic compensation and vascular tone.

#### 4.3.1. Sedation, Psychomotor Impairment, and Respiratory Compromise

The best-documented acute pharmacodynamic interactions involve central nervous system depressants, particularly opioids, benzodiazepines and sedative agents [53]. More broadly, controlled evidence also indicates clinically relevant alcohol interactions with several CNS-active drugs, including diazepam, opioids, methylphenidate and cannabis [10]. At higher exposures, the most serious medical consequence is respiratory compromise. This is particularly notable with opioids, which exert respiratory depressant effects manifested as suppression of ventilatory control and reduced responsiveness to hypercapnia and hypoxia [54,55].

A systematic review and meta-analysis of 107 placebo-controlled studies including 3097 participants identified the clearest controlled interaction signals for diazepam, opioids, methylphenidate and cannabis. Alcohol either increased peak drug exposure or enhanced safety-relevant CNS effects, although mechanisms differed between drug classes [10]. Similar observations have been made in more recent controlled studies of newer centrally acting agents. Alcohol coadministration increased lemborexant exposure and additively impaired cognition, worsened the cognitive effects of zuranolone compared with zuranolone alone, and enhanced psychomotor impairment with sunobinop without any major pharmacokinetic interaction [56,57,58]. Together, these findings show that ethanol can increase CNS toxicity, either by raising exposure to selected drugs or by exacerbating their effects on vigilance, cognition, psychomotor performance and respiratory control.

The clinical relevance of this problem is highlighted by recent real-world data. Patients with alcohol use disorder often experience potential alcohol–medication interactions which frequently involve psychoactive drugs, analgesics and cardiovascular drugs; hence, polypharmacy and comorbidity are associated with an elevated risk of pharmacodynamic interaction [11]. In response, the POSAMINO criteria were developed through expert consensus to identify potentially serious alcohol-drug interactions in older adults. These criteria were used to classify CNS-related alcohol-drug interactions in a prospective cohort study of 1457 community-dwelling adults aged ≥ 65 years; the interactions classified as potentially serious based on these criteria were found to be associated with a higher risk of falls [9,12,13,59].

Among these outcomes, the most serious are impaired airway protection and ventilatory drive, particularly during opioid co-exposure [54,55].

A controlled dose-escalating study in opioid-naïve volunteers showed that ethanol enhanced oxycodone-induced respiratory depression and increased apneic events, with greater vulnerability in older participants [60]. Experimental data also suggest that ethanol–benzodiazepine interactions may include a pharmacokinetic component in addition to the well-established pharmacodynamic CNS depressant effect, although such animal data should be interpreted as evidence supporting biological plausibility support rather than direct clinical evidence [61].

Jedeikin et al. report the case of a patient who was admitted with combined benzodiazepine and alcohol exposure [62]. The patient entered a coma and developed acute respiratory failure. Interestingly, after apparent awakening, the patient continued to demonstrate an impaired ventilatory response to carbon dioxide, suggesting that the respiratory center recovered more slowly than the level of consciousness [62]. Indeed, combined exposure to alcohol, benzodiazepines and other psychotropic or sedative drugs has been associated with a greater risk of impaired driving-related performance, severe intoxication and fatal poisoning, particularly when several CNS depressants are used concurrently [63,64,65].

In a retrospective forensic study of 380 decedents from Spain, medical psychotropics were detected in 42.4% of cases, polypharmacy in 25.0%, and ethanol in more than one-third; in addition, about one-third of psychotropic users had also been exposed to ethanol [66]. The study also found sex-related differences: in men combined exposure was more common, and psychotropic use was associated with a lower blood alcohol concentration. This finding suggests that measured blood alcohol concentration may underestimate the functional risk of combined psychotropic and ethanol exposure.

#### 4.3.2. Delayed Hypoglycemia and Metabolic Decompensation

**Figure 2:**
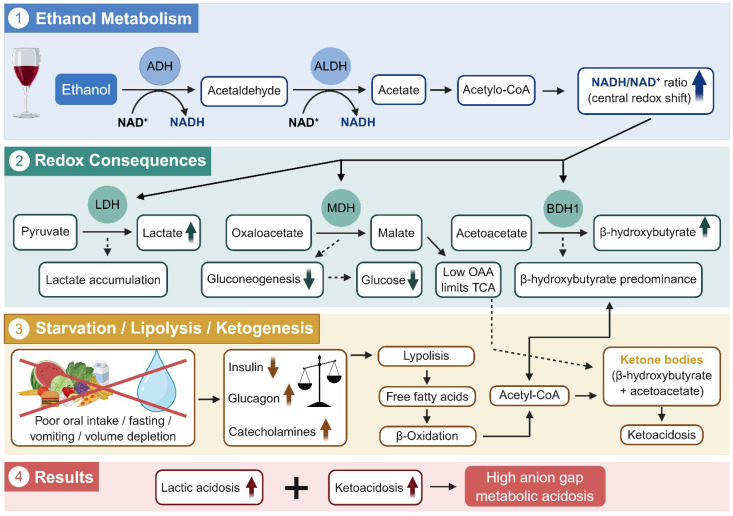
Schematic overview of the metabolic mechanisms leading to alcoholic acidosis. Ethanol metabolism increases the NADH/NAD^+^ ratio, thereby promoting lactate formation, suppressing gluconeogenesis, reducing oxaloacetate availability for the TCA cycle, and favoring β-hydroxybutyrate predominance. In addition, fasting, vomiting and volume depletion stimulate lipolysis and ketogenesis. The combined effect results in lactic acidosis, ketoacidosis, and high-anion-gap metabolic acidosis [16,69]. ADH, alcohol dehydrogenase; ALDH, aldehyde dehydrogenase; LDH, lactate dehydrogenase; MDH, malate dehydrogenase; BDH1, β-hydroxybutyrate dehydrogenase 1; NAD^+^, oxidized nicotinamide adenine dinucleotide; NADH, reduced nicotinamide adenine dinucleotide; OAA, oxaloacetate; TCA, tricarboxylic acid cycle; acetyl-CoA, acetyl coenzyme A. Created with BioRender.com. Bukowska, B. https://BioRender.com/97xlbe2 (accessed on 5 July 2026).

The interaction between acute ethanol exposure and glucose-lowering therapy may be misleading in practice because patients can initially appear stable but develop delayed hypoglycemia the following morning. For example, in a crossover study, six men with type 1 diabetes received standardized meals and insulin, as well as evening wine intake equivalent to 0.75 g/kg ethanol. While no significant change was observed in overnight glucose profiles, fasting and post-breakfast glucose were lower the next day, and five participants required treatment for hypoglycemia between 10:00 a.m. and noon [67]. Richardson et al. later reinforced the same clinical message in 16 free-living adults with type 1 diabetes monitored for 36 h with CGMS. This was important because it extended the delayed-hypoglycemia signal beyond laboratory conditions and showed that the “next-morning” risk remained relevant in everyday life [68]. Metformin is characterized by a distinct type of interaction: in susceptible settings, the principal concern is not hypoglycemia but lactic acidosis. Ethanol oxidation increases the hepatic NADH/NAD+ ratio, shifts pyruvate toward lactate and suppresses gluconeogenesis, which may further impair hepatic lactate utilization in metformin-treated patients (Figure 2). This risk is likely to be greatest during heavy acute alcohol intake accompanied by fasting, vomiting, dehydration, renal impairment or hepatic dysfunction [69].

In metformin-treated patients, this redox effect may become important for patient safety because metformin already limits hepatic glucose production and may further impair lactate handling, especially when renal perfusion is reduced. The result is a phenotype that differs from delayed morning hypoglycemia observed after alcohol: the dominant risk is lactate accumulation and metabolic acidosis rather than isolated glucose decline. The greatest risk is associated with the combination of reduced hepatic gluconeogenesis and impaired lactate clearance, particularly during acute alcohol intake accompanied by fasting, dehydration, hypotension, renal impairment or reduced renal perfusion [16,69,70]. Notably, Miyata et al. reported a case of metformin-associated lactic acidosis in a 66-year-old man who took metformin after consuming a relatively small amount of alcohol, i.e., approximately 700 mL of beer [70]. However, this observation should only be interpreted as a clinical signal rather than proof that modest alcohol intake consistently precipitates metformin-associated lactic acidosis. Similar case-based literature indicates that excessive or binge drinking may precipitate metformin-associated lactic acidosis, especially when coexisting with renal impairment, dehydration, fasting, reduced oral intake or alcoholic ketoacidosis [69,71]. Overall, alcohol should be viewed as a contextual amplifier of metformin-associated lactic acidosis, particularly when fasting, dehydration, renal impairment or hepatic dysfunction coexist. The interaction with sodium-glucose cotransporter 2 (SGLT2) inhibitors should not be interpreted as a classic direct pharmacokinetic or pharmacodynamic ethanol–drug interaction. Rather, alcohol may act as a contextual risk amplifier when alcohol-related fasting, vomiting, dehydration or reduced carbohydrate intake overlaps with established triggers of euglycemic diabetic ketoacidosis. SGLT2 inhibitor-associated ketoacidosis was first formally recognized after reports of euglycemic diabetic ketoacidosis in treated patients; it is now understood as a drug-related metabolic phenotype that can be precipitated by catabolic stressors such as fasting, acute illness, reduced carbohydrate intake, dehydration, surgery or insulin deficiency [72,73,74,75,76]. Recent reviews and case reports emphasize that SGLT2 inhibitor-associated ketoacidosis may occur with normal or only modestly elevated glucose levels, and that its recognition may be delayed by alcohol use or suspected alcoholic ketoacidosis [77,78,79,80]. Therefore, alcohol exposure should be considered a contextual risk amplifier for metabolic decompensation in patients receiving glucose-lowering therapy, particularly when reduced oral intake, dehydration, acute illness or renal impairment is present.

The main controlled human studies and informative case-based reports supporting ethanol-related pharmacodynamic interactions in the central nervous system, respiratory control, cognition, and glucose/lactate homeostasis are summarized in Table 3.

#### 4.3.3. Orthostatic and Hemodynamic Instability

Cardiovascular effects should be interpreted with caution. In ethanol–drug interactions, the best-supported cardiovascular phenotype is hemodynamic instability rather than direct cardiotoxicity. Acute alcohol exposure can potentiate orthostatic hypotension and enhance the hypotensive effects of drugs that lower blood pressure or weaken compensatory vasoconstriction, including vasodilators such as prazosin and felodipine [83,84,85]. In a double-blind, randomized, placebo-controlled study of 14 healthy young volunteers, Narkiewicz et al. showed that alcohol doubled the systolic blood pressure fall during −40 mmHg lower-body negative pressure, from −7 to −14 mmHg, and prevented the usual rise in forearm vascular resistance [84]. Kawano et al. reported a similar interaction patter in 10 Japanese men with mild hypertension. Participants received 1 mL/kg alcohol with a light meal before and after five to seven days of prazosin treatment at 1 mg three times daily, with ambulatory blood pressure monitoring every 30 min for 24 h. Blood pressure fell two to four hours after drinking, and this fall was greater during prazosin treatment, changing from −18.0 ± 3.7/−11.8 ± 2.7 mmHg before prazosin to −24.4 ± 4.9/−17.8 ± 2.8 mmHg during prazosin treatment [85].

A randomized, crossover, double-blind study by Bailey et al. identified a similar interaction with felodipine in 10 patients with untreated borderline hypertension. After a non-intoxicating dose of ethanol or placebo, followed by felodipine 5 mg, the maximal hemodynamic effect occurred after four hours. Compared with felodipine alone, the combination reduced supine total peripheral resistance to 13 ± 2 vs. 17 ± 2 mmHg/L/min, lowered diastolic blood pressure to 68 ± 3 vs. 75 ± 2 mmHg, and increased heart rate to 72 ± 3 vs. 67 ± 2 beats/min. The fall in standing blood pressure was even more pronounced, reaching 113 ± 8/69 ± 5 mmHg compared to 126 ± 5/82 ± 3 mmHg with felodipine alone [83]. In a randomized crossover study, co-administration of 10 mg TPN171 with 0.5 g/kg alcohol in healthy men significantly lowered the 0–4 h systolic blood pressure effect curve compared with TPN171 alone and significantly increased pulse-rate exposure. The combination also produced a greater maximal rise in heart rate compared with alcohol alone, although neither agent measurably altered the pharmacokinetics of the other [86]. Similarly, alcohol co-administration increased systemic tunodafil exposure in healthy men, with AUC_0–∞_ and C_max_ rising by 42.89% and 74.46%, respectively. Pharmacodynamically, alcohol remained the dominant factor lowering blood pressure, whereas PDE5 inhibitor contributed to an additive increase in heart rate [87]. For patients prone to hypotension, the clinical consequences may include dizziness, flushing, palpitations, syncope, falls, or poor tolerance of otherwise routine vasodilator therapy [8,59]. The nifedipine study illustrates that the same clinical phenotype may also reflect a mixed pharmacokinetic and hemodynamic interaction, because ethanol increased nifedipine exposure and shortened the time to peak heart-rate response [47]. Together, these newer vasodilator studies reinforce the concept that ethanol-related hemodynamic interactions are primarily pharmacodynamic; however, they may also be accompanied by drug-specific pharmacokinetic changes that increase the likelihood of dizziness, flushing, reflex tachycardia or syncope in susceptible patients [86,87].

Representative controlled human studies on acute ethanol-related hemodynamic effects and interactions with antihypertensive or vasodilatory drugs are summarized in Table 4.

#### 4.3.4. Disulfiram and Disulfiram-like Reactions

**Figure 3:**
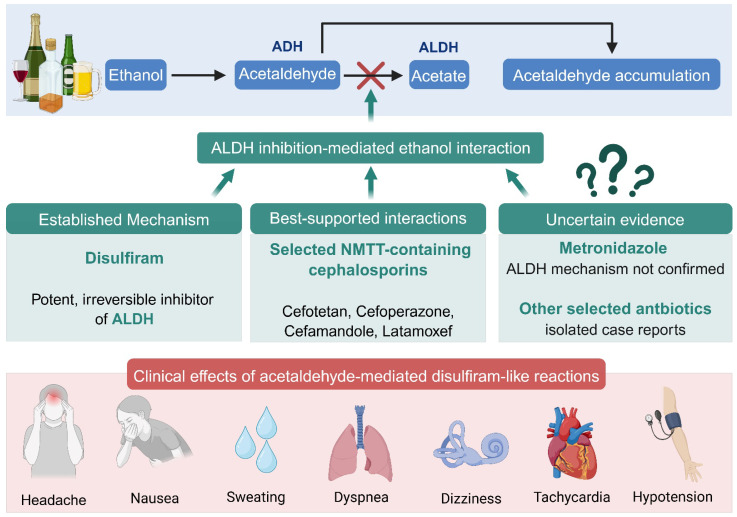
Ethanol reactions associated with ALDH inhibition: the strongest evidence exists for disulfiram, followed by selected NMTT-containing cephalosporins. The evidence for metronidazole remains weak or inconclusive. Created with BioRender.com. Bukowska, B. https://BioRender.com/lrqwdb7 (accessed on 5 July 2026).

The clearest model in this field remains the disulfiram–ethanol reaction. When ALDH is inhibited by disulfiram, acetaldehyde accumulates rapidly after alcohol intake, producing flushing, nausea, palpitations, vomiting, hypotension and, in severe cases, circulatory collapse [88]. Disulfiram-like reactions have also been reported for selected cephalosporins, particularly those containing an *N*-methylthiotetrazole (NMTT) side chain, such as cefamandole, cefoperazone, cefotetan and latamoxef. In a cefotetan challenge study, five of eight healthy volunteers developed obvious flushing after ethanol exposure, accompanied by an increase in heart rate [89]. By contrast, no comparable findings were observed in 15 volunteers receiving cefonicid, indicating that this risk is structure-dependent rather than a class effect of all cephalosporins [88,90] (Figure 3).

The evidence for metronidazole is weaker and remains debated. In a double-blind study, Visapää et al. found no increase in acetaldehyde accumulation and no objective or subjective disulfiram-like response after ethanol challenge in healthy men receiving metronidazole [91]. A recent reappraisal similarly concluded that the evidence for a meaningful metronidazole–ethanol interaction is weak, with isolated case reports and older observations contrasted by several negative clinical datasets [92]. This distinction is important because traditional warnings often group these reactions together, although the strength of clinical evidence differs markedly between disulfiram, selected NMTT-containing cephalosporins and metronidazole [93]. Therefore, disulfiram and selected NMTT-containing cephalosporins warrant high-confidence warnings during alcohol exposure, whereas metronidazole should be presented as a lower-certainty and debated interaction [88,89,90,91,92,93]. Low-certainty signals have also been described for other nitroimidazoles, selected antibiotics and non-antibiotic products. For example, a recent case report described flushing, nausea, vomiting, tachycardia and hypotension after alcohol intake during ornidazole therapy, although the mechanism was not confirmed [94]. Pharmacovigilance data indicate that drug-associated alcohol intolerance continues to be reported, but these reports should be interpreted as hypothesis-generating signals rather than proof of an ALDH-mediated mechanism [95]. Recent cephalosporin case reports, including reactions during cefmetazole and cefoperazone–sulbactam therapy, support a structure- and context-dependent interpretation rather than broad antibiotic–alcohol warnings [96,97]. Exposure to non-beverage ethanol during disulfiram therapy may also elicit important adverse reactions, as illustrated by intolerance after the use of alcohol-containing hand sanitizers [98]. Severe hemodynamic instability and fatal outcomes have been reported in high-risk patients after disulfiram–ethanol exposure [99,100]. These reports reinforce high-confidence warnings for disulfiram itself, but should not be extrapolated to all traditionally suspected disulfiram-like drugs. Evidence for ketoconazole, griseofulvin, trimethoprim/sulfamethoxazole and isolated herbal products remains limited or inconclusive, and current reviews do not indicate that commonly cited antibiotics including oral penicillin, cefdinir, cefpodoxime, fluoroquinolones, azithromycin, tetracyclines or fluconazole exhibit predictable disulfiram-like reactions [88]. Isolated case reports also suggest that disulfiram-like reactions may occasionally be described with non-antibiotic and herbal products. Dang et al. reported the case of a 32-year-old man who developed vomiting, palpitations, and flushing after alcohol intake while taking Ginaton (*Ginkgo biloba* extract) 80 mg twice daily for one week. This observation is noteworthy, but the evidence remains very weak because it is based on a single case report and the proposed acetaldehyde-based mechanism was not directly confirmed [101].

The human evidence supporting established, absent or uncertain disulfiram-like reactions after ethanol exposure is summarized in Table 5.

### 4.4. Acute Organ-Level Toxicity

**Figure 4:**
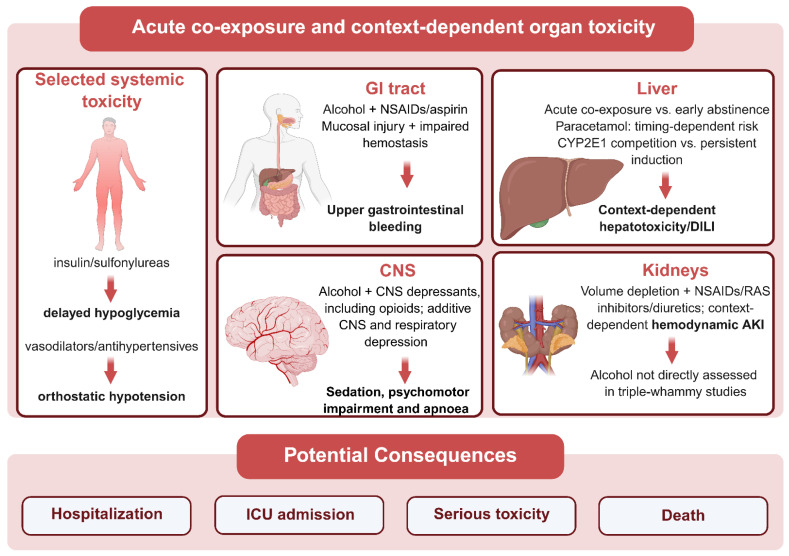
Major clinical consequences of ethanol–drug interactions. Created with BioRender.com. Bukowska, B. https://BioRender.com/9gb3xvy (accessed on 5 July 2026).

For some ethanol–drug pairs, the main clinical outcome is organ injury resulting from overlapping pharmacodynamic, pharmacokinetic and patient-specific vulnerability factors. Ethanol may increase tissue susceptibility, alter drug exposure or reactive-metabolite burden, and reduce the capacity to compensate for oxidative, inflammatory or hemodynamic stress [16,33,102]. This can result in upper gastrointestinal bleeding, drug-induced liver injury (DILI), or acute kidney injury (AKI). The following sections distinguish direct evidence of acute ethanol–drug toxicity from indirect or context-dependent evidence involving habitual alcohol use, recent abstinence or additional physiological stressors. Importantly, the strength of causal evidence differs between organ systems. Alcohol is supported by epidemiological data and biological evidence as a cofactor in NSAID- or aspirin-associated upper gastrointestinal bleeding [102,103,104]. For paracetamol/acetaminophen hepatotoxicity, risk is strongly dependent on timing and clinical context. Short-term therapeutic-dose exposure appears to carry limited risk in controlled studies of newly abstinent alcohol-dependent patients. In contrast, greater concern applies to selected high-risk states, including recent alcohol cessation after repeated drinking, malnutrition, prolonged paracetamol/acetaminophen use, or impaired hepatic reserve [16,23,24,46]. For acute kidney injury, evidence is more indirect and mainly supports a multi-hit scenario involving dehydration, renal hypoperfusion, NSAID exposure, RAS blockade, diuretics or pre-existing renal vulnerability [25,26,27,105]. This concept is summarized in Figure 4.

#### 4.4.1. Gastrointestinal Bleeding During Alcohol and NSAID/Aspirin Exposure

In the gastrointestinal tract, ethanol increases mucosal permeability and vulnerability to injury through both local and systemic mechanisms. Aspirin and other NSAIDs, especially COX-1 inhibitors, reduce synthesis of protective prostaglandins, weaken the gastric mucus–bicarbonate barrier, impair microvascular homeostasis, and, in the case of aspirin, inhibit platelet aggregation. Alcohol and NSAIDs therefore do not simply coexist as separate risk factors: they can amplify one another at the level of epithelial injury and impaired hemostatic leading to overt upper gastrointestinal bleeding (UGIB) [102,106]. Not all NSAIDs demonstrate the same baseline bleeding risk; alcohol may further increase this risk particularly with regular NSAID/aspirin use, higher alcohol intake, or both. In a meta-analysis of 25 studies, celecoxib was found to have the lowest pooled risk of gastrointestinal bleeding, with an odds ratio (OR) of 1.16. Among nonselective NSAIDs, the lowest risk was demonstrated by ibuprofen (OR 2.28), which was still significant, and the highest risk was observed for ketorolac (OR 20.67) [107]. These estimates describe NSAID-specific baseline gastrointestinal bleeding risk and should not be interpreted as alcohol-specific effect estimates. From a therapeutic safety perspective, the bleeding risk associated with alcohol is superimposed on an NSAID-specific baseline bleeding risk, which can differ markedly between agents. A Saskatchewan Health case–control analysis comparing 1083 hospitalizations for severe gastrointestinal events with 14,754 controls indicated that NSAID exposure alone and a history of alcohol abuse each carried an odds ratio of 2.9, whereas their coexistence was associated with an odds ratio of 10.2 [103]. These estimates suggest a supra-additive pattern of gastrointestinal risk when alcohol misuse and NSAID exposure coexist. Similarly, Kaufman et al. report that heavy alcohol intake independently increased the risk of acute major UGIB, with the highest incidence associated with concurrent use of aspirin or ibuprofen [106]. Among current drinkers, regular ibuprofen use was associated with an estimated relative risk (RR) of 2.7. This is consistent with data from male regular NSAID/aspirin users, in whom the risk of gastrointestinal bleeding increased with alcohol intake, with a multivariable RR of 1.75 for ≥15 g/day compared with non-drinkers [104].

#### 4.4.2. Acute Kidney Injury During Alcohol and NSAID Co-Exposure

Ethanol-related kidney injury (AKI) is best interpreted as a context-dependent, multi-hit phenotype rather than as a classic direct nephrotoxic drug interaction [25,105]. The most relevant case reports describe the occurrence of reversible flank-pain-associated AKI after combined binge alcohol intake and NSAID exposure in young adults, which can sometimes require temporary hemodialysis [108,109,110,111,112]. These cases are biologically plausible because alcohol-related volume depletion, vomiting, reduced intake or hypotension may lower renal perfusion, while NSAIDs impair prostaglandin-dependent afferent arteriolar vasodilation [25,105]. Renal elimination may also become important for drug safety relevant as an indirect consequence of ethanol-related hemodynamic instability rather than as a specific tubular transporter interaction. Acute alcohol intake may promote transient diuresis and is often accompanied by vomiting, reduced oral intake or poor fluid replacement, thereby lowering effective circulating volume and renal perfusion. In patients receiving NSAIDs, RAS inhibitors or diuretics, this may support the formation of a hemodynamic status characterized by compromised afferent prostaglandin-dependent vasodilation, efferent arteriolar tone and circulating volume [26,27,113]. Alcohol was not directly assessed in the major “triple-whammy” pharmacoepidemiological studies; however, these data support a broader model in which alcohol-related dehydration, vomiting, reduced oral intake, hypotension, orthostatic hypotension or acute illness-related renal hypoperfusion may act as additional hemodynamic stressors and situational amplifiers of AKI risk in susceptible patients exposed to NSAIDs, RAS inhibitors and/or diuretics. Under such conditions, impaired renal elimination may increase exposure to renally eliminated drugs such as lithium and metformin. [69,114]. Thus, for gastrointestinal bleeding, the evidence includes direct epidemiological data on alcohol combined with aspirin or NSAIDs, whereas for kidney injury the evidence mainly supports an indirect, multi-hit hemodynamic model. The relevant case reports, case series and indirect pharmacoepidemiological evidence are summarized in Table 6.

#### 4.4.3. Timing-Dependent Acute Hepatotoxicity

For acute ethanol–drug co-exposure, the risk of hepatotoxicity should be evaluated in relation to timing rather than ethanol exposure alone. One important example is paracetamol/acetaminophen, for which ethanol-related risk depends on whether ethanol is present during drug metabolism or whether paracetamol/acetaminophen exposure occurs after repeated or chronic alcohol intake. During acute co-exposure, ethanol may act mainly as a transient metabolic competitor, whereas the period following repeated or chronic alcohol exposure is characterized by a different metabolic state in which adaptive changes may persist [16,23,46]. As such, the temporal window of greatest concern may not necessarily coincide with peak blood ethanol concentration, but occur after ethanol has been cleared; during this later period, the liver may be more susceptible to injury due to hepatic metabolic adaptation, reduced nutritional reserve, concurrent illness or impaired hepatic reserve [23,115,116,117]. Detailed mechanisms involving chronic exposure, CYP2E1 induction, NAPQI formation, glutathione reserve and therapeutic-dose paracetamol/acetaminophen studies in abstinent alcohol-dependent patients are discussed in Section 5.5.

## 5. Effects of Chronic Alcohol Exposure on Drug Disposition and Toxicity

Chronic alcohol exposure produces a pharmacological state distinct from acute ethanol–drug co-exposure. Rather than transient metabolic competition, long-term exposure may remodel hepatic drug-metabolizing enzymes and transporters, disrupt intestinal barrier integrity and, in advanced alcohol-associated liver disease, reduce hepatic functional reserve. These effects must also be distinguished from those occurring during early abstinence and from the consequences of established alcohol-associated liver disease.

### 5.1. Chronic Remodeling of Drug-Metabolizing Enzymes

**Figure 5:**
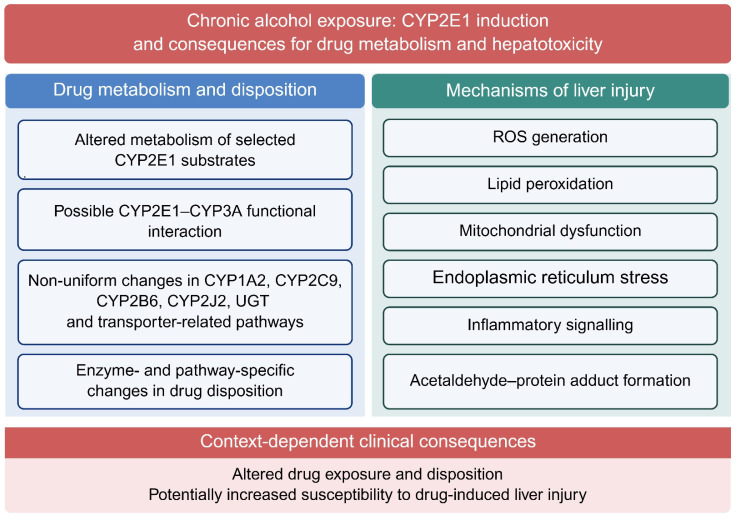
The effect of chronic alcohol exposure on altered drug disposition, drug bioactivation and increased susceptibility to hepatotoxicity via CYP2E1-centered mechanisms. Chronic ethanol exposure induces CYP2E1 activity and may also remodel other hepatic drug-metabolizing enzymes and transporter-related pathways. Created with BioRender.com. Bukowska, B. https://BioRender.com/z7nqi36 (accessed on 5 July 2026).

As such, the temporal window of greatest concern may not necessarily coincide with peak blood ethanol concentration, but may occur after ethanol has been cleared, particularly when adaptive metabolic changes persist; during this later period, the liver may be more susceptible to injury due to hepatic metabolic adaptation, reduced nutritional reserve, concurrent illness or impaired hepatic reserve. Indeed, human pharmacokinetic, phenotyping and human-liver protein data indicate that chronic alcohol use may affect several drug-metabolizing pathways, including CYP1A2, CYP2C9, CYP2C19, CYP2D6, CYP2B6, CYP2J2, CYP3A-dependent activity and selected UGT-related pathways. These effects are dependent on a range of factors, such as enzyme type, substrate, drinking pattern and liver status [18,20,33,34]. Nevertheless, CYP2E1 still plays a central part because its activity is induced, and stabilized, by repeated ethanol exposure, increasing microsomal ethanol oxidation and ROS generation during catalytic cycling. Such CYP2E1-derived oxidative stress may promote reactive-metabolite formation, lipid peroxidation, mitochondrial dysfunction, endoplasmic reticulum stress, acetaldehyde- and protein-adduct formation, glutathione depletion and inflammatory signaling [118,119,120]. These processes can lower the hepatic threshold for drug-induced injury through their influence on impaired mitochondrial β-oxidation, reduced antioxidant reserve, gut-derived inflammatory stimuli and alcohol-associated liver disease. This risk is particularly apparent in cases characterized by drug bioactivation, fasting, malnutrition, acute illness or reduced hepatic reserve. These mechanisms are summarized in Figure 5.

Hepatic drug metabolism is, then, remodeled by chronic alcohol consumption. This remodeling is both pathway-specific and network-dependent, as confirmed by recent ex vivo findings. While chronic ethanol exposure is classically associated with CYP2E1 induction, its pharmacokinetic effect may extend beyond the direct metabolism of CYP2E1 substrates. This has important clinical value, because CYP3A enzymes, particularly CYP3A4, metabolize a much broader spectrum of therapeutic agents than CYP2E1 [121].

A study of human liver microsomes obtained from 23 donors with documented differences in alcohol exposure found chronic alcohol exposure to be associated with increased CYP3A-dependent metabolism of two substrates: 7-benzyloxyquinoline and ivermectin. Importantly, CYP3A activity correlated with CYP2E1 abundance rather than with CYP3A4/CYP3A5 protein levels [121]. This is a significant finding as CYP2E1 shows only negligible activity toward 7-benzyloxyquinoline and does not metabolize ivermectin; hence, this increased metabolism of CYP3A substrates is unlikely to result from direct CYP2E1-mediated catalysis [121]. Instead, these findings suggest that the microsomal cytochrome P450 system operates as an integrated multienzyme network. In this network, individual P450 isoforms compete for NADPH-cytochrome P450 reductase, a common electron donor, and form heteromeric protein complexes that modify catalytic function. Indeed, physical CYP2E1-CYP3A4 interactions have been identified by tag-transfer chemical crosslinking mass spectrometry, and data suggests that CYP2E1 may alter CYP3A4 function through protein–protein interactions within the endoplasmic reticulum membrane [121].

Therefore, long-term CYP2E1 upregulation caused by chronic alcohol use may influence CYP3A-dependent metabolism in a substrate- and context-dependent manner, but these ex vivo findings should not be interpreted as uniform clinical induction of CYP3A activity. As a result, part of the CYP3A4 pool may be shifted from less active or “latent” configurations toward more catalytically competent states. As CYP3A4 is involved in the metabolism of many drugs, such indirect activation of CYP3A broadens the pharmacokinetic consequences of chronic alcohol exposure to more than direct CYP2E1 substrates [121].

More detailed human liver proteomic data support this broader view. In heavy drinkers, CYP2E1 protein abundance increased 1.7-fold and its contribution to the hepatic P450 pool rose from 12.9% to 23.0%, whereas CYP1A2 and CYP2C9 abundance decreased to 43% and 67% of non-drinker levels, respectively. Alcohol exposure was also associated with altered glucuronidation-related protein abundance, including lower UGT1A4, UGT2B7, UGT2B10 and UGT2B15, relatively higher UGT1A6 and UGT1A9, and higher MRP3 abundance [18]. These findings support the view that chronic alcohol exposure redistributes hepatic drug-handling capacity across phase I enzymes, phase II pathways and export mechanisms rather than simply inducing CYP2E1.

In practical terms, chronic alcohol exposure should not be expected to shift all drugs in the same direction; different substrates may show increased, decreased or less predictable clearance depending on the metabolic pathways that dominate their disposition.

### 5.2. Drug Handling During Early Abstinence and Withdrawal

The period of early abstinence following chronic alcohol exposure should be considered a transitional pharmacological state rather than complete metabolic recovery. In this period, ethanol is no longer present as an acute metabolic competitor; however, some consequences of repeated alcohol exposure may persist, including altered enzyme activity, nutritional depletion, reduced glutathione reserve, withdrawal-related physiological stress and changes in hepatic drug-handling capacity [16,23,24,115,116]. Therefore, early abstinence should not be treated as either active ethanol co-exposure or a fully normalized metabolic condition. Indeed, no consistent biochemical liver injury was found to be associated with therapeutic-dose paracetamol/acetaminophen use in studies involving recently abstinent alcohol-dependent patients [23,24,115,116]. While these findings should not be extrapolated to overdose, malnutrition, acute illness or advanced liver disease, they nevertheless indicate that the risk associated with paracetamol/acetaminophen depends strongly on timing, dose and host reserve.

Drug activity and exposure may also change in specific ways during early abstinence. In six patients withdrawing from chronic alcohol use, fixed-dose chlordiazepoxide therapy (six days) was associated with falling, rather than increasing, plasma concentrations of chlordiazepoxide and desmethylchlordiazepoxide [122]. A similar timing-dependent pattern was observed for carbamazepine: alcohol-dependent patients exhibited higher carbamazepine concentrations and lower carbamazepine-10,11-epoxide concentrations after heavy alcohol exposure than after nine days of abstinence. This difference suggests transient metabolic inhibition during intoxication and greater metabolite formation after ethanol withdrawal [123].

This finding is borne out by recent literature on the pharmacotherapy associated with alcohol withdrawal. One prospective pharmacokinetic study showed poor correlation between cumulative dose, plasma concentrations and clinical effects in patients after chlordiazepoxide treatment for alcohol withdrawal symptoms; as such, chlordiazepoxide exposure and clinical response may be difficult to predict during withdrawal [124]. This variability is particularly relevant when hepatic dysfunction is present. In patients with alcohol-associated liver disease, it is recommended that benzodiazepine treatment should be based on agents less dependent on oxidative hepatic metabolism, especially lorazepam [125]. A recent review found chlordiazepoxide to pose a potential risk of prolonged sedation in hepatic insufficiency due to dose-stacking caused by delayed onset and its active metabolites [126].

However, it should be noted that the available studies are generally small and fail to fully identify the underlying mechanisms; as such, these findings should be interpreted with caution. Nevertheless, they show that during withdrawal or early abstinence, drug exposure may change over only a few days, even under supervised conditions. Therefore, when assessing drug safety, early abstinence should therefore be considered as a discrete stage and unlike acute ethanol–drug co-exposure or stable chronic alcohol exposure.

### 5.3. Hepatic Transporter Remodeling During Chronic Alcohol Exposure and Alcohol-Associated Cirrhosis

Chronic alcohol exposure is associated with changes in hepatic drug-metabolizing enzymes and transporter-related proteins [18]. However, most direct evidence concerning individual hepatic uptake and efflux transporters is derived from liver tissue obtained from patients with alcohol-associated cirrhosis rather than from studies of uncomplicated chronic alcohol exposure. Studies based on targeted quantitative proteomics have demonstrated reduced hepatic abundance of the uptake transporters OATP1B1 and OATP1B3 and increased abundance of the basolateral efflux transporter MRP3 in alcohol-associated cirrhosis [38]. A reduced abundance of OATP1B1/OATP1B3 may decrease hepatic uptake of susceptible drug substrates, whereas increased levels of MRP3 may enhance their efflux from hepatocytes into the systemic circulation. Other transporters showed smaller, variable or non-significant changes, indicating transporter-specific remodeling rather than generalized suppression of hepatic transport. These findings suggest that transporter remodeling may contribute to altered and less predictable drug exposure in alcohol-associated cirrhosis irrespective of changes in CYP-mediated metabolism. Nevertheless, because these data were obtained from cirrhotic liver tissue, they should not be extrapolated directly to all individuals with chronic alcohol consumption.

### 5.4. Chronic Intestinal Injury and the Gut–Liver Axis

Chronic heavy alcohol exposure may disrupt intestinal barrier integrity, alter the composition and metabolic activity of the gut microbiota, and increase intestinal permeability. Human studies have demonstrated increased permeability to macromolecules and endotoxemia in patients with chronic alcohol misuse, although these abnormalities do not occur uniformly in all alcohol-dependent individuals [127,128]. Barrier disruption facilitates the translocation of microbial products, including lipopolysaccharide, into the portal circulation. These products may contribute to the development and progression of alcohol-associated liver disease through the activation of hepatic inflammatory pathways. This gut–liver feedback loop may be further reinforced by dysbiosis, intestinal inflammation and acetaldehyde-mediated epithelial injury.

These chronic intestinal changes could theoretically modify oral drug absorption, luminal drug metabolism and intestinal first-pass handling by influencing intestinal permeability, luminal conditions, barrier integrity and mucosal inflammatory tone [129]. However, direct clinical evidence linking alcohol-associated dysbiosis or increased intestinal permeability to altered exposure to specific drugs remains limited. Accordingly, the gut–liver axis should currently be presented primarily as a modifier of intestinal and hepatic vulnerability, rather than as a firmly established mechanism of clinically significant pharmacokinetic interactions.

### 5.5. Pharmacokinetic Consequences of Alcohol-Associated Liver Disease

Ethanol itself is not a classic displacement agent for most drugs, but chronic alcohol use can still alter their distribution indirectly, particularly through advanced alcohol-associated liver disease or cirrhosis. The resulting hypoalbuminemia and hyperbilirubinemia, and qualitative changes in albumin binding, increase the free fraction of drugs with high protein binding. Early studies in patients with chronic alcoholism or alcohol-associated liver disease revealed altered plasma protein binding profiles for diazepam, salicylate, sulfadiazine, phenylbutazone and valproate [130,131,132]. Brodie and Boobis indicate that reduced binding is not a uniform consequence of alcohol exposure itself: salicylate, sulphadiazine, and phenylbutazone binding remained normal in chronic drinkers without overt liver disease, but decreased in those with alcoholic liver disease. The degree of binding also correlated with bilirubin and albumin in alcoholic hepatitis [130]. Thiessen et al. also report lower binding of diazepam in patients with chronic alcohol consumption, with binding values of 97.8 ± 1.2% versus 98.5 ± 0.4% for diazepam under the reported assay conditions [132]. Similar results have been noted for valproate at 80 μg/mL, whose binding fell from 88.7 ± 5.2% in controls to 70.7 ± 11.3% in alcoholic cirrhosis [131]. This finding has important safety implications because the free fraction may increase even when total plasma concentrations appear therapeutic. Furthermore, total concentrations may differ from free concentrations, which can be higher than expected; as a result, reliance on total drug levels alone may underestimate the degree of pharmacologically active exposure. This risk is greatest for drugs with a narrow therapeutic index, and in patients with reduced physiological reserve [130,131,132,133]. Indeed, more recent data on cirrhosis confirm a mismatch between total concentrations and free drug concentrations for drugs with high protein binding affinity; in addition, recent valproate data further support direct free-drug monitoring when binding is disrupted [35,133]. Alcohol-associated cirrhosis may also decrease first-pass extraction and systemic clearance by reducing hepatic blood flow and causing portosystemic shunting; this loss would be particularly significant for drugs with a high hepatic extraction ratio. Loss of functional hepatocyte mass and impaired biliary elimination may further increase drug exposure [37]. The loss of metabolic reserve is not uniform across enzymatic pathways. A clinical phenotyping study found advanced cirrhosis reduced activity markers for CYP1A2, CYP2B6, CYP2C19, CYP2D6 and CYP3A, without any significant change in CYP2C9 activity [36]. These findings indicate that drug clearance in advanced liver disease becomes pathway- and disease-severity-dependent rather than uniformly reduced. Taken together, hypoalbuminemia, hyperbilirubinemia, impaired hepatic perfusion, portosystemic shunting and loss of functional metabolic capacity may increase free drug exposure and make clearance less predictable in alcohol-associated liver disease. These consequences reflect established liver dysfunction rather than a direct effect of ethanol itself. Older clinical pharmacokinetic studies reported increased drug clearance and increased tolbutamide metabolism in patients with alcohol dependence [134,135]. The tolbutamide data are relevant because tolbutamide is primarily cleared by CYP2C9-dependent hydroxylation rather than by CYP2E1; this implies that chronic alcohol exposure may also affect non-CYP2E1 P450 pathways, including CYP2C9 [134]. Older studies also indicate that tetracycline/doxycycline, diazepam, chlordiazepoxide and thiopentone exhibit altered pharmacokinetics in chronic alcohol users [136,137,138,139]. However, while these findings support the hypothesis that drug exposure and metabolism may be influenced by the type of substrate during chronic alcohol exposure, they should not be interpreted as evidence of uniform CYP induction. The principal human and human-tissue evidence concerning chronic alcohol-related remodeling of hepatic enzymes and transporters, as well as the pharmacokinetic consequences of alcohol-associated liver disease, is summarized in Table 7 and Table 8.

### 5.6. Chronic Alcohol Exposure, Abstinence and Drug-Induced Liver Injury

Chronic alcohol exposure may increase vulnerability to paracetamol/acetaminophen hepatotoxicity through CYP2E1 induction and increased generation of *N*-acetyl-p-benzoquinone imine (NAPQI). This risk is modified by nutritional status and hepatic antioxidant capacity: fasting and malnutrition may limit NAPQI detoxification by reducing hepatic glutathione availability [16,115,116,117]. The APAP toxicity sequence involves metabolic activation to NAPQI, followed by glutathione depletion, covalent protein adduct formation, mitochondrial oxidant stress and necrotic hepatocyte death; the process is mediated by CYP2E1. The sequence can be modified by alcohol at several context-dependent points: chronic exposure may increase CYP2E1-dependent bioactivation. In addition, fasting or malnutrition, which may accompany harmful alcohol use, early abstinence, vomiting or poor oral intake, may reduce hepatic glutathione reserves and thereby limit NAPQI detoxification, increasing susceptibility to APAP-induced liver injury. In addition, acute co-exposure may result in alcohol transiently competing for CYP2E1-mediated metabolism. Therefore, the modifying effects of alcohol consumption on paracetamol/acetaminophen hepatotoxicity are likely to depend on timing, dose and host state and should not be interpreted as a uniform risk factor [115,116,144].

Controlled trials in newly abstinent alcohol-dependent patients did not show short-term worsening of hepatic injury markers during therapeutic-dose acetaminophen exposure. Kuffner et al. found no increase in AST or ALT after acetaminophen 1 g four times daily for two days [145], and a larger randomized study including 443 subjects found similar peak ALT values after acetaminophen 4 g/day for three days compared with placebo [24]. Bartels et al. similarly reported no significant increase in AST, ALT or INR during short-term sustained-release acetaminophen treatment in recently abstinent alcohol-dependent patients [146]. By contrast, case reports and clinical series describe severe hepatotoxicity in selected regular drinkers, particularly after recent alcohol cessation, malnutrition or prolonged/repeated dosing [23,147,148]. Thus, early abstinence should not be treated as either active ethanol co-exposure or complete metabolic recovery. Therapeutic-dose paracetamol/acetaminophen is not uniformly hazardous under controlled abstinence conditions; however, the risk may be elevated in cases of persistent CYP2E1 induction, loss of ethanol-mediated competitive inhibition, fasting, malnutrition, reduced glutathione availability, repeated supratherapeutic dosing or acute illness.

Drug-induced liver injury has also been associated with other medications. In a meta-analysis of 53 studies, alcohol consumption increased the risk of liver injury following anti-tuberculosis drug use, with a pooled odds ratio of 1.55 (95% CI 1.19–2.04) [149]. A more recent two-center cohort study also identified baseline alcohol use as one of the pretreatment predictors of anti-tuberculosis drug-induced liver injury, supporting closer liver-function monitoring in patients with alcohol exposure before therapy [28].

A similar exposure-dependent relationship has been reported for methotrexate. In a CPRD cohort of 11,839 patients with rheumatoid arthritis starting methotrexate, alcohol intake above 21 units per week was associated with a higher risk of transaminitis, i.e., more than three times the upper limit of normal, with an adjusted hazard ratio of 1.85 (95% CI 1.17–2.93); however, intake below 14 units per week was not associated with any increased risk at that endpoint [150]. Hence, alcohol should be treated as a dose- and context-dependent, modifiable cofactor of hepatotoxicity that lowers the threshold for DILI, rather than directly initiating it, particularly in patients with liver disease, malnutrition or polypharmacy. Overall, susceptibility to DILI during chronic alcohol exposure and early abstinence depends on the drug involved, drinking pattern, timing of alcohol cessation, nutritional status, glutathione reserve and severity of underlying liver disease. Representative evidence is summarized in Table 9.

## 6. Cross-Pattern and Multifactorial Ethanol–Drug Interactions

Some ethanol–drug interactions cannot be assigned to a single exposure pattern because their clinical expression depends on timing, chronic metabolic adaptation, organ reserve, adherence, diet and concomitant medications. These cross-pattern interactions are clinically important because the same drug may carry different risks during acute co-exposure, after repeated or chronic alcohol use, during early abstinence, or in alcohol-associated liver disease.

Three examples are especially useful for clinical interpretation: paracetamol/acetaminophen, disulfiram-like reactions and warfarin. Paracetamol/acetaminophen illustrates a timing-dependent interaction, because acute ethanol co-exposure may differ mechanistically from paracetamol/acetaminophen exposure after repeated or chronic alcohol intake or during early abstinence [23,24].

In contrast, disulfiram and selected NMTT-containing cephalosporins interfere with ALDH-dependent acetaldehyde clearance, thereby increasing acetaldehyde accumulation, whereas the traditional warning associated with metronidazole remains more controversial and less consistently supported by controlled human evidence [88,89,91,93].

Finally, warfarin illustrates a multifactorial alcohol–drug interaction in which changes in anticoagulation cannot be reduced to a single pharmacokinetic mechanism [153,154].

### Warfarin and Unstable Anticoagulation

The influence of alcohol exposure on warfarin is difficult to interpret because anticoagulation may be destabilized through several pathways at once. Acute alcohol intake may transiently increase the anticoagulant response, whereas chronic heavy drinking may contribute to hepatic dysfunction and greater variability of warfarin response. Alcohol-related liver injury may also reduce hepatic synthesis of clotting factors and clotting reserve independently of warfarin exposure, while binge drinking may worsen adherence, alter diet and vitamin K intake, and increase trauma risk [153,154].

In one case, a 58-year-old man with previously stable warfarin control developed an increased international normalized ratio (INR) after beginning regular low-dose beer consumption, with anticoagulation returning to baseline after abstinence [153]. As a single case report, this observation should be interpreted as a signal of possible INR destabilization rather than as proof that low-dose alcohol consistently increases warfarin effects. Its main value is to illustrate that alcohol-related changes in anticoagulation may occur even outside overt heavy drinking, especially when additional factors affecting diet, adherence, liver function and anticoagulant sensitivity are present.

A later community-based case–control study of 570 warfarin users, including 265 with major bleeding and 305 controls, linked moderate or severe alcohol misuse with major bleeding (OR 2.10; 95% CI 1.08–4.07), and heavy episodic drinking, defined as five or more drinks on one occasion, with an OR of 2.36 (95% CI 1.24–4.50) [154]. Therefore, the interaction between warfarin and alcohol should not be viewed simply as INR variability, but as a clinically significant bleeding-risk scenario shaped by drinking pattern, liver function, diet, adherence, trauma risk and concomitant medications.

## 7. Conclusions

Ethanol–drug interactions are best understood as mechanism-based and context-dependent events whose clinical importance depends on the timing and pattern of alcohol exposure.

1. Acute ethanol exposure most often amplifies pharmacodynamic toxicity and disturbs early pharmacokinetic processes.
2. In contrast, chronic exposure, early abstinence and especially advanced alcohol-associated liver disease are more strongly associated with CYP2E1 induction, broader hepatic enzyme and transporter remodeling, altered protein binding, reduced organ reserve and greater susceptibility to liver or kidney injury.
3. Recurring mechanisms of ethanol modulation include CNS additivity, ethanol oxidation with NADH/NAD^+^ redox shift, altered first-pass handling, CES1- and CYP-mediated metabolism, formulation-dependent dose dumping, impaired hepatic or renal compensation, and ALDH inhibition with acetaldehyde accumulation in disulfiram-like reactions.
4. The strength of causal evidence differs between endpoints, with the most direct human evidence supporting interactions with CNS depressants, selected controlled pharmacokinetic/pharmacodynamic interactions, formulation-specific alcohol-induced dose dumping, and ethanol-related modification of glucose and hemodynamic responses.
5. For organ toxicity, alcohol has relatively direct support as a cofactor in NSAID- or aspirin-associated upper gastrointestinal bleeding. The paracetamol/acetaminophen example further illustrates that ethanol should not be treated as a uniform hepatotoxic cofactor, but as a timing-, nutrition- and liver-status-dependent modifier of drug-induced injury. Acute kidney injury, in contrast, is best framed as an indirect multi-hit hemodynamic model.
6. Risk assessment should therefore consider drinking pattern, timing, dosage form, organ reserve, early abstinence, alcohol-associated liver disease, dehydration or acute illness, polypharmacy and over-the-counter drug use.

## Figures and Tables

**Table 1:** Ethanol effects on modified-release formulations, gastric emptying, and early absorption.

Interacting Agents	Design, Sample, and Evidence Category	Main Finding	Clinical Interpretation	Study or Report
Hydrocodone ER 50 mg with 20% or 40% alcohol	Open-label randomized crossover alcohol-interaction study (*n* = 30 naltrexone-blocked healthy volunteers); controlled human evidence	20% alcohol did not meaningfully alter hydrocodone exposure. With 40% alcohol, AUC increased by about 20%, C_max_ increased approximately 2.3-fold, and t_max_ shortened from 6.16 to 2.43 h; no complete dose dumping was observed.	High-concentration alcohol may accelerate early absorption and increase peak opioid exposure even when the ER formulation does not fully dose dump.	[41]
Ethanol (240 mL of 40% alcohol) with morphine ER (KADIAN)	Open-label randomized crossover study (*n* = 32 healthy men); controlled human evidence	In 32 healthy men, concomitant intake of alcohol with morphine ER did not meaningfully alter morphine C_max_ or AUC under fed or fasted conditions.	Alcohol-induced dose dumping is formulation-specific and was not observed with this morphine ER product.	[42]
Oxycodone prolonged-release tablets with 240 mL of 20% or 40% alcohol	Phase I open-label randomized crossover study (*n* = 20 healthy volunteers); controlled human evidence	No alcohol-induced dose dumping was observed. Once-daily oxycodone showed a slight increase in overall absorption with 40% alcohol but no increase in peak absorption; twice-daily oxycodone showed peak and overall increases with 40% alcohol but retained a prolonged-release profile.	Alcohol-induced dose dumping is formulation-specific. Even when dose dumping is not observed, alcohol should be avoided with opioids because of increased exposure and pharmacodynamic CNS/respiratory risk.	[43]
Amphetamine extended-release orally disintegrating tablet with alcohol	Randomized single-dose open-label four-period crossover study (*n* = 32 randomized; 27 completed); controlled human evidence	Alcohol up to 40% did not meaningfully alter amphetamine bioavailability, and the extended-release profile was retained.	This study shows that some ER stimulant formulations may remain robust in the presence of alcohol, reinforcing that alcohol effects on modified-release products are formulation-dependent.	[44]
300 mL of 4% or 10% ethanol solution, beer, or red wine with a high-calorie solid meal	Human ultrasonographic study (*n* = 16 healthy men; high- and low-calorie meal subgroups, *n* = 8 each); controlled human evidence	For the high-calorie solid meal subgroup, gastric emptying half-time was 131.3 ± 7.0 min with water and increased to 158.8 ± 9.3 min with 4% ethanol, 165.6 ± 6.2 min with 10% ethanol, 163.1 ± 11.0 min with beer and 186.3 ± 8.4 min with red wine. The lag phase was not significantly changed versus water (48.1 ± 6.5 min).	Alcohol may delay gastric emptying and shift the timing of drug absorption.	[45]

**Table 2:** Acute ethanol-related modifiers of drug metabolism and first-pass disposition: pathway-selective CYP inhibition and CES1-mediated interactions.

Interacting Agents	Design, Sample, and Evidence Category	Main Finding	Clinical Interpretation	Study or Report
Acute metabolic competition: CYP phenotyping and pathway-selective inhibition				
Acute ethanol (0.7 g/L) with phenotyping cocktail: caffeine 150 mg, tolbutamide 125 mg, omeprazole 20 mg, dextromethorphan 30 mg, oral midazolam 2 mg, intravenous midazolam 1 mg, and digoxin 0.5 mg	Randomized crossover probe-drug cocktail study (*n* = 16 healthy volunteers); controlled human evidence	In 16 healthy volunteers, acute ethanol exposure increased dextromethorphan AUC_0_–t 1.95-fold and caffeine AUC_0_–t 1.38-fold. Intestinal first-pass extraction of midazolam decreased to 77% of control, whereas CYP2C9, CYP2C19, NAT2, and P-gp probe outcomes were not meaningfully affected.	Acute ethanol produces pathway-selective metabolic effects rather than broad inhibition of drug metabolism and transport.	[17]
Acute ethanol (0.8 g/kg) with CYP2E1 probe chlorzoxazone (500 mg)	Human chlorzoxazone phenotyping study/landmark CYP2E1 probe study (ethanol arm *n* = 7; diallyl sulfide arm *n* = 8); controlled human evidence	Acute ethanol reduced the 6-hydroxychlorzoxazone/chlorzoxazone ratio, an in vivo CYP2E1 phenotyping index.	Acute ethanol may transiently inhibit CYP2E1 activity during co-exposure; this should be distinguished from CYP2E1 induction after chronic alcohol exposure.	[46]
CES1-mediated and first-pass metabolic interactions				
Ethanol (0.6 g/kg) with (0.3 mg/kg) dl-methylphenidate or (0.15 mg/kg) d-methylphenidate	Randomized crossover study (*n* = 24 healthy volunteers); controlled human evidence	Ethanol increased early exposure to the active d-methylphenidate enantiomer by 44–99% during absorption after racemic methylphenidate dosing and increased total d-methylphenidate AUC by 21%; this is consistent with altered CES1-mediated first-pass metabolism.	Ethanol may increase active d-methylphenidate exposure by altering CES1-mediated first-pass metabolism.	[32]
Ethanol with dexmethylphenidate or dl-methylphenidate SODAS formulations	Open-label crossover study in healthy volunteers (*n* = 14 completed); controlled human evidence	Ethanol altered methylphenidate exposure in a formulation- and enantiomer-dependent manner, with relevance to early exposure and ethylphenidate formation.	The ethanol-methylphenidate interaction depends on formulation, enantiomer composition, and CES1-mediated first-pass handling.	[52]
Ethanol target BAC 0.08 g/dL with oseltamivir 150 mg or aspirin 650 mg	Human pharmacokinetic study in healthy volunteers (*n* = 18), supported by in vitro CES data; controlled human evidence/supporting biological evidence	Alcohol increased oseltamivir AUC_0–6_ h by 27% and decreased the oseltamivir carboxylate/oseltamivir AUC_0–6_ h ratio by 34%, consistent with inhibition of CES1-mediated oseltamivir hydrolysis. Aspirin pharmacokinetics were not meaningfully affected.	Ethanol can inhibit CES1-mediated hydrolysis in humans, but the effect is pathway- and substrate-selective and may not extend to CES2-mediated aspirin hydrolysis.	[21]

**Table 3:** Ethanol-related pharmacodynamic interactions affecting CNS function, respiration, cognition, and metabolic control.

Interacting Agents	Design, Sample, and Evidence Category	Main Finding	Clinical Interpretation	Study or Report
Central nervous system depressants, cognitive/psychomotor impairment, and respiratory compromise				
Ethanol with benzodiazepines	Case report (*n* = 1); case-report evidence	Coma, acute respiratory failure, and prolonged impairment of the ventilatory response to CO_2_ after apparent clinical recovery.	Delayed recovery of ventilatory control after CNS depressant co-exposure.	[62]
Oxycodone 20 mg with ethanol target concentrations of 0, 0.5, or 1.0 g/L	Dose-escalating experimental study in opioid-naïve volunteers (*n* = 24; 12 young and 12 elderly participants); controlled human evidence	Oxycodone reduced baseline minute ventilation by 28%. Ethanol produced an additional reduction in minute ventilation; it thereby enhanced oxycodone-induced respiratory depression and increased apneic events, with greater vulnerability in older participants.	Alcohol can potentiate opioid-induced respiratory depression, supporting strict avoidance of alcohol during opioid use, especially in older adults.	[60]
Alcohol–medication interactions involving diazepam, opioids, methylphenidate and cannabis	Systematic review and meta-analysis of 107 placebo-controlled studies (*n* = 3097 participants)	Controlled interaction signals were identified for diazepam, opioids, methylphenidate and cannabis. Alcohol either increased peak drug exposure or enhanced safety-relevant central nervous system effects, although mechanisms differed across drug classes.	Selected ethanol–drug interactions are reproducible under controlled conditions and may involve either pharmacokinetic exposure changes or pharmacodynamic CNS additivity.	[10]
Amitriptyline 25 mg with acute ethanol co-exposure targeting blood ethanol concentration of approximately 0.8 g/L	Pharmacokinetic/pharmacodynamic study in healthy volunteers (*n* = 5); amitriptyline 25 mg with ethanol or juice; ethanol given 1 h before amitriptyline and continued for 8 h; controlled human evidence	Ethanol increased early free amitriptyline exposure: free plasma concentrations rose by 204%, 186% and 127% at 1.5, 2 and 2.5 h, respectively; free AUC_0–8_ h increased by 48 ± 13% (*p* < 0.01). Postural sway increased by 92% with ethanol versus 2% without ethanol, and word recall decreased by 71% versus 37%.	Acute ethanol may increase free amitriptyline exposure and enhance psychomotor and cognitive impairment; alcohol should be avoided during therapy.	[81]
Lemborexant 10 mg with ethanol 0.7 g/kg in men and 0.6 g/kg in women	Randomized double-blind crossover study (*n* = 32 healthy adults); controlled human evidence	Ethanol increased lemborexant AUC by 70% and C_max_ by 35% and additively impaired cognitive performance.	Mixed PK/PD interaction: increased lemborexant exposure plus additive CNS impairment.	[58]
Zuranolone 50 mg with ethanol 0.7 g/kg in men and 0.6 g/kg in women	Randomized double-blind crossover study in healthy adults (*n* = 25); controlled human evidence	Zuranolone alone produced small-to-moderate cognitive impairment, whereas ethanol produced stronger impairment. Co-administration generally worsened cognitive performance compared with zuranolone alone.	Mainly pharmacodynamic CNS interaction: alcohol may add to cognitive impairment during zuranolone treatment.	[56]
Brivaracetam 200 mg with ethanol target blood concentration 0.6 g/L	Randomized crossover study (*n* = 18 healthy men); controlled human evidence	Brivaracetam did not meaningfully alter ethanol pharmacokinetics, but enhanced alcohol-related psychomotor and cognitive impairment.	Pharmacodynamic CNS additivity without major PK interaction.	[82]
Sunobinop (centrally active/investigational agent) 2 or 6 mg with alcohol 0.7 g/kg	Randomized double-blind placebo-controlled crossover study in healthy adults (*n* = 48); controlled human evidence	Alcohol alone impaired psychomotor/cognitive performance up to 2 h, whereas sunobinop produced dose-dependent impairment. Combined sunobinop and alcohol caused greater impairment than either treatment alone up to 8 h, without a notable PK interaction; somnolence was the most common adverse event.	Alcohol may add to CNS/psychomotor impairment with centrally active NOP receptor partial agonists, even when a major pharmacokinetic interaction is not evident.	[57]
Potential alcohol–medication interactions in patients with alcohol use disorder	Real-world addiction-ward study; observational clinical evidence	Potential alcohol–medication interactions were common and frequently involved psychoactive drugs, analgesics, glucose-lowering drugs, and selected cardiovascular drug classes.	Pharmacodynamic interaction risk often occurs in the setting of polypharmacy, comorbidity and alcohol use disorder.	[11]
Glucose-lowering drugs and metabolic decompensation				
Evening alcohol with insulin-treated type 1 diabetes	Controlled crossover study (*n* = 6 men with type 1 diabetes; ethanol 0.75 g/kg as wine); controlled human evidence	Blood ethanol peaked at 19.1 ± 1.2 mmol/L and was undetectable by 08:00. There were no evening/overnight glucose differences, but the post-breakfast glucose peak was lower after wine than water (8.9 ± 1.7 vs. 15.0 ± 1.5 mmol/L). Hypoglycemia required treatment in 5/6 subjects between 10:00 and 12:00, with nadirs of 1.9–2.9 mmol/L; 0/6 required treatment after water.	This finding illustrates delayed post-alcohol hypoglycemia, characterized by stable overnight glucose but reduced post-breakfast glucose and late-morning episodes requiring treatment.	[67]
Ethanol with insulin-treated type 1 diabetes	Free-living CGMS study (*n* = 16 adults with type 1 diabetes; 36 h monitoring); observational clinical evidence/prospective monitoring study	Sixteen adults with type 1 diabetes (age 39 ± 7 years; diabetes duration 15 ± 11 years; HbA1c 8.1 ± 1.0%) wore CGMS for 36 h. After evening alcohol intake, interstitial glucose was lower on the following day, supporting delayed hypoglycemia risk in real-life conditions.	This finding confirms delayed post-alcohol glucose lowering under real-life conditions in type 1 diabetes.	[68]
Metformin 1500 mg/day with modest alcohol intake (~700 mL beer; ~28 g ethanol)	Case report (*n* = 1; 66-year-old man receiving metformin)	After approximately 700 mL of beer, the patient presented with shock and metabolic acidosis: blood pressure 68/43 mmHg, arterial pH 7.33, bicarbonate 16.2 mmol/L and lactate 7.4 mmol/L. Vasopressor support was discontinued after 12 h.	In this patient, modest alcohol intake was temporally associated with MALA, but the report does not establish a general causal relationship.	[70]
SGLT2 inhibitors with alcohol-related fasting, vomiting, dehydration or reduced carbohydrate intake	Case reports and reviews/contextual clinical signal	SGLT2 inhibitor-associated ketoacidosis may occur at normal or only modestly elevated glucose levels. Alcohol use or suspected alcoholic ketoacidosis may delay its recognition.	Alcohol should be interpreted as a contextual risk amplifier rather than as a classic ethanol-SGLT2 inhibitor interaction, especially when reduced intake, dehydration, acute illness or renal impairment is present.	[78,79,80]

**Table 4:** Acute ethanol-related hemodynamic effects and interactions with antihypertensive and vasodilatory drugs.

Interacting Agents	Design, Sample, and Evidence Category	Main Finding	Clinical Interpretation	Study or Report
Ethanol (1 g/kg) with orthostatic stress	Randomized placebo-controlled physiological study (*n* = 14); controlled human evidence	During –40 mmHg lower-body negative pressure, alcohol approximately doubled the systolic blood pressure fall (–14 vs. –7 mmHg) and attenuated reflex vasoconstriction.	This finding supports alcohol-related amplification of orthostatic hypotension and fall risk.	[84]
Prazosin 1 mg three times daily with alcohol 1 mL/kg	Controlled repeated-measures study in 10 Japanese men with mild essential hypertension; ambulatory BP measured every 30 min for 24 h before and after 5–7 days of prazosin treatment; controlled human evidence	Alcohol reduced blood pressure for several hours. This reduction was enhanced by prazosin 2–4 h after intake; BP fell from −18.0 ± 3.7/−11.8 ± 2.7 mmHg before treatment to −24.4 ± 4.9/−17.8 ± 2.8 mmHg during prazosin treatment.	α_1_-Adrenergic blockade may impair compensatory vasoconstriction and accentuate acute alcohol-induced hypotension in patients treated with prazosin.	[85]
Felodipine (5 mg) with a non-intoxicating ethanol dose	Randomized crossover study (*n* = 10 patients with untreated borderline hypertension); controlled human evidence	In 10 patients, felodipine plus ethanol produced greater blood pressure reduction than felodipine alone, especially standing BP (113/69 vs. 126/82 mmHg), with increased heart rate (72 ± 3 vs. 67 ± 2 bpm).	This study supports ethanol-related amplification of felodipine-induced hypotension and reflex tachycardia.	[83]
Ethanol (75 mL of 94% in orange juice) with nifedipine (20 mg)	Randomized crossover PK/PD study (*n* = 10 healthy volunteers); controlled human evidence	Ethanol increased nifedipine AUC by 54% (533 vs. 346 ng·h/mL), while C_max_, t_max_ and half-life were not significantly changed. Time to peak heart-rate response was shortened from 2.2 to 1.4 h.	This finding supports ethanol-related increase in nifedipine exposure and acceleration of the heart-rate response.	[47]
TPN171 10 mg with ethanol 0.5 g/kg	Randomized crossover study in healthy Chinese men (*n* = 15); controlled human evidence	TPN171 plus alcohol lowered the 0–4 h systolic blood pressure effect curve versus TPN171 alone and increased pulse-rate exposure; alcohol did not alter TPN171 pharmacokinetics and TPN171 did not alter alcohol pharmacokinetics.	The interaction was mainly pharmacodynamic, with greater pulse-rate response but no major PK interaction. The interaction was mainly pharmacodynamic, with greater pulse-rate response but no major PK interaction.	[86]
Ethanol (0.5 g/kg) with tunodafil (100 mg)	Randomized blinded placebo-controlled three-cycle crossover study (*n* = 18 healthy men); controlled human evidence	In 18 healthy men, co-administration with alcohol increased tunodafil AUC_0–∞_ by 42.89% and C_max_ by 74.46%; metabolite M459 AUC_0–∞_ and C_max_ increased by 28.75% and 39.32%, respectively.	Alcohol-related increase in tunodafil exposure may enhance vasodilator-related adverse effects.	[87]

**Table 5:** Disulfiram and disulfiram-like reactions after ethanol exposure.

Interacting Agents	Design, Sample, and Evidence Category	Main Finding	Clinical Interpretation	Study or Report
Disulfiram with ethanol exposure	Single case report (*n* = 1); clinical signal	Acute ethanol–disulfiram reaction presented with marked hemodynamic instability.	Supports high-confidence warnings for disulfiram itself during ethanol exposure.	[99]
Disulfiram with ethanol exposure in a patient with pre-existing cardiac comorbidities	Single fatal case report (*n* = 1); clinical signal	Fatal disulfiram–ethanol reaction occurred in a high-risk patient with cardiac comorbidities.	Illustrates that disulfiram–ethanol reactions may be life-threatening or fatal in susceptible patients.	[100]
Cefmetazole with alcohol exposure	Single case report (*n* = 1); clinical signal	Disulfiram-like reaction was described after alcohol exposure during cefmetazole therapy.	Supports the existence of a structure-dependent risk among selected cephalosporins rather than a uniform cephalosporin class effect.	[96]
Cefoperazone-sulbactam with accidental alcohol exposure	Single pediatric case report (*n* = 1); clinical signal	Severe disulfiram-like reaction occurred in a child after accidental alcohol ingestion during cefoperazone-sulbactam therapy.	Suggests possible NMTT-related alcohol intolerance, based on a single case report.	[97]
Ornidazole with alcohol intake	Single case report (*n* = 1); clinical signal	Flushing, nausea, vomiting, tachycardia and hypotension occurred after alcohol intake during ornidazole therapy.	Suggests that alcohol intolerance may occasionally occur with other nitroimidazoles, but causality and mechanism remain unconfirmed.	[94]
Cefotetan 2 g × 3 doses at 12 h intervals with ethanol 0.5 g/kg	Ethanol challenge study (*n* = 8 healthy volunteers); controlled human evidence	Ethanol challenge after cefotetan exposure produced flushing in 5 of 8 volunteers and was associated with an increase in heart rate.	Cefotetan may produce a medically relevant disulfiram-like reaction after alcohol exposure, consistent with selected NMTT-containing cephalosporins.	[89]
Cefonicid 1 g single dose or 1 g × 3 doses at 24 h intervals with ethanol challenge	Placebo-controlled study (*n* = 15 healthy volunteers; single and multiple 1 g cefonicid doses plus ethanol); controlled human evidence	Single cefonicid dosing and three consecutive 1 g doses at 24 h intervals followed by ethanol challenge did not produce a disulfiram-type reaction. Blood acetaldehyde concentrations did not increase.	Lack of reaction with cefonicid supports structure-dependent, rather than class-wide, disulfiram-like risk among cephalosporins.	[90]
Metronidazole for 5 days with ethanol 0.4 g/kg challenge	Double-blind controlled study (*n* = 12 healthy men); controlled human evidence	In 12 healthy men, 5 days of metronidazole followed by ethanol challenge did not raise blood acetaldehyde concentrations and no clinically measurable disulfiram-like response was observed.	Controlled human data do not support a disulfiram-like reaction with metronidazole under the tested conditions.	[91]
*Ginkgo biloba* extract (Ginaton) 80 mg twice daily with alcohol intake	Single case report (*n* = 1); clinical signal	A patient developed flushing, palpitations, and vomiting after alcohol intake during Ginaton use, suggesting a possible disulfiram-like reaction.	This case suggests a possible Ginaton–alcohol reaction, but causality remains unconfirmed.	[101]

**Table 6:** Alcohol-related gastrointestinal bleeding and renal injury during pharmacotherapy.

Interacting Agents	Design, Sample, and Evidence Category	Main Finding	Clinical Interpretation	Study or Report
NSAID-associated gastrointestinal bleeding				
Alcohol intake ≥ 21 drinks/week with aspirin use (>325 mg every other day, lower-dose regular use, or occasional use) or regular ibuprofen use	Interview-based case–control study of acute major UGIB; 1224 hospitalized cases and 2945 controls; observational clinical evidence	Compared with <1 drink/week, acute UGIB risk increased with alcohol intake and reached RR 2.8 at ≥21 drinks/week. Among current drinkers, UGIB risk was higher with aspirin use, particularly regular aspirin >325 mg every other day, and with regular ibuprofen use.	Alcohol may increase gastrointestinal bleeding risk when combined with aspirin or regular NSAID use.	[106]
History of alcohol abuse with NSAIDs	Saskatchewan Health case–control analysis (1083 hospitalized GI-event cases; 14,754 controls); observational clinical evidence	NSAID exposure alone and alcohol misuse alone were each associated with increased GI-event risk (OR 2.9 for both). Combined NSAID exposure and alcohol misuse showed a much higher risk (OR 10.2).	Combined alcohol misuse and NSAID exposure may markedly increase gastrointestinal event risk beyond either factor alone.	[103]
Alcohol consumption with regular NSAID and/or aspirin use	Prospective cohort study in men (*n* = 48,000; 26-year follow-up); observational clinical evidence	During 883,797 person-years of follow-up, 305 major GIB episodes were confirmed. Among regular NSAID/aspirin users, GIB risk increased with alcohol intake; for ≥15 g/day alcohol, multivariable RR was 1.75 (95% CI, 1.07–2.88) versus non-drinkers.	Alcohol may potentiate NSAID/aspirin-associated gastrointestinal bleeding risk, particularly with regular use and higher alcohol intake.	[104]
Renal perfusion and acute kidney injury				
Binge alcohol intake with NSAID ingestion	Case reports/short case series in young adults after binge drinking and NSAID use	Reversible AKI with back/flank pain occurred after binge alcohol intake and NSAID ingestion, without overt rhabdomyolysis.	These cases support a multi-hit mechanism: alcohol-related volume depletion or hypoperfusion combined with NSAID-mediated impairment of renal prostaglandin-dependent vasodilation.	[109,110,112]
Binge drinking followed by NSAID use	Case report (*n* = 1); clinical signal	After binge drinking and subsequent NSAID use, the patient developed anuria and severe AKI; creatinine rose from 5.4 to 11.6 mg/dL, and temporary hemodialysis was required.	Alcohol-related volume depletion followed by NSAID exposure may cause reversible but dialysis-requiring AKI.	[111]
Ethanol and/or NSAID exposure in acute flank pain syndrome	Retrospective Icelandic case series of young adults with AKI and flank pain (*n* = 21; median age 26 years, range 19–35); clinical signal	Acute flank pain syndrome accounted for 21/106 AKI cases. NSAID intake and ethanol consumption were each reported in 15 patients, and both exposures in 9 patients. Symptoms resolved over days to weeks.	This series supports reversible flank-pain-associated AKI as a recognizable phenotype in young adults, often linked to NSAID use, ethanol intake, or both.	[108]
Diuretic plus ACE inhibitor/ARB and NSAID, with alcohol considered as a potential additional hemodynamic stressor	Nested case–control study in antihypertensive users; cohort *n* = 487,372; AKI cases *n* = 2215; controls *n* = 21,993; observational clinical evidence; indirect for alcohol-related AKI	During 5.9 ± 3.4 years of follow-up, AKI incidence was 7/10,000 person-years. Triple therapy with a diuretic, ACE inhibitor/ARB, and NSAID increased AKI risk, particularly within the first 30 days, while double therapy did not.	Alcohol was not directly tested, but alcohol-related dehydration, hypotension, or renal hypoperfusion may add to this hemodynamic AKI risk in susceptible patients.	[27]
NSAID use with RAS inhibitor and/or diuretic therapy	Nested case–control study in community users of RAS inhibitors and/or diuretics (*n* = 78,379); observational clinical evidence; indirect for alcohol-related AKI	NSAID exposure increased AKI risk in patients receiving RAS inhibitors and/or diuretics, with the greatest absolute excess risk observed in triple therapy.	Alcohol was not directly tested, but alcohol-related volume depletion or hypotension may further increase hemodynamic AKI risk in this setting.	[26]

**Table 7:** Chronic alcohol exposure as a modifier of hepatic drug-metabolizing enzymes in non-cirrhotic or human-liver experimental settings.

Interacting Agents	Design, Sample, and Evidence Category	Main Finding	Clinical Interpretation	Study or Report
Alcohol-related CYP2E1 enrichment and CYP3A4 function	In vitro study using human liver microsomes from heavy alcohol consumers and controls; supporting biological evidence	Human liver microsomes from heavy alcohol consumers showed higher fractional CYP2E1 content and altered CYP3A4 functional behavior compared with controls.	Chronic alcohol exposure may affect drug metabolism both through CYP2E1 induction and possible CYP3A4 functional modulation.	[20]
Chronic heavy alcohol exposure + hepatic DMET proteins	Human liver microsome study (*n* = 94 donors; chronic alcohol exposure based on donor history, not controlled ethanol dosing); supporting biological evidence; supporting biological evidence	Chronic alcohol exposure altered CYP2E1, CYP2B6, CYP2J2, selected UGTs and transporter-related proteins. CYP2E1 abundance increased ~1.7-fold, its share in the hepatic P450 pool increased from 12.9% to 23.0%, whereas CYP1A2 and CYP2C9 decreased to 43% and 67% of non-drinker levels. MRP3 abundance increased.	Chronic alcohol exposure may remodel hepatic drug-metabolizing enzymes and transporters in a pathway-specific manner, rather than causing generalized induction.	[18]
Chronic alcohol exposure, CYP2E1 abundance and CYP3A function	Human liver microsomes from 23 donors with different levels of alcohol exposure; no controlled ethanol dose; supporting biological evidence; preprint	CYP3A-dependent metabolism increased with alcohol exposure and correlated with CYP2E1 abundance rather than CYP3A protein levels. CYP2E1-CYP3A4 complexes were also identified.	CYP2E1 induction may modify CYP3A activity through protein–protein interactions, although clinical relevance remains uncertain.	[121]
CYP2E1-related oxidative liver injury	Reviews focusing on biological mechanisms	CYP2E1-related oxidative stress may promote lipid peroxidation, mitochondrial dysfunction, ER stress, acetaldehyde/protein adduct formation and inflammatory signaling.	These mechanisms may lower the threshold for drug-induced liver injury, especially during malnutrition, fasting, glutathione depletion, acute illness or alcohol-associated liver disease.	[119,120]
Chlordiazepoxide during fixed-dose alcohol-withdrawal therapy	Pharmacokinetic observation in six patients with chronic heavy alcohol use: >80 g ethanol/day for ≥10 years; age 21–56 years; chlordiazepoxide 25 mg orally every 6 h for 6 days; human pharmacokinetic evidence	Chlordiazepoxide and desmethyl chlordiazepoxide concentrations were significantly lower on day 6 than on day 2. Demoxepam continued to increase, with marked interindividual variability.	Early abstinence and withdrawal may represent a dynamic pharmacokinetic state. Apparent clearance of chlordiazepoxide and desmethylchlordiazepoxide may increase during withdrawal.	[122]
Amitriptyline biotransformation in depressive patients with alcohol dependency	Clinical pharmacokinetic study in depressive inpatients at steady state: 11 non-alcoholic and 10 alcohol-dependent patients; human pharmacokinetic evidence	Patients with alcohol dependency demonstrated lower amitriptyline demethylation, but higher conjugation and hydroxylation.	Chronic alcohol-related clinical status may alter antidepressant biotransformation in a pathway-specific manner. The study predates modern CYP phenotyping.	[140]
Methadone metabolism after acute and chronic ethanol exposure	Rat in vivo and liver microsome study after acute or chronic ethanol exposure; animal and in vitro study	Chronic ethanol decreased tissue methadone concentrations and increased microsomal *N*-demethylation. Acute ethanol increased brain and liver methadone concentrations and inhibited microsomal *N*-demethylation.	Ethanol-methadone interactions may be timing-dependent: acute exposure may inhibit metabolism, whereas chronic exposure may enhance microsomal degradation.	[141]
Heroin and methadone hepatotoxicity after ethanol pretreatment	In vitro study using cultured human hepatocytes pretreated with ethanol 50 or 100 mM and then exposed to increasing concentrations of heroin or methadone	Ethanol pretreatment potentiated opioid-induced hepatotoxicity. TC50 decreased by ~55–70% for heroin and ~40–60% for methadone. Urea synthesis, glycogen metabolism and intracellular glutathione were impaired. Cytochrome P450 levels increased by ~40%.	Ethanol exposure may increase opioid-related hepatocellular vulnerability through higher P450 content and glutathione depletion.	[142]
Phenytoin during long-term alcohol use and withdrawal	Clinical pharmacokinetic study in alcohol-dependent men with withdrawal seizures, *n* = 11; clearance analysis in 9/11; ethanol 20% *v*/*v* for 6 days; phenytoin 150 mg every 12 h for 20 days; human pharmacokinetic evidence	Mean phenytoin clearance increased from 0.023 ± 0.006 to 0.033 ± 0.013 L/kg/h during withdrawal. Steady-state concentrations ranged from 3.4 to 29.9 mg/L. Bioavailability varied little: 0.93–1.03. Free fractions ranged from 9.09% to 17.75%; total and free concentrations correlated strongly.	Withdrawal may reveal enzyme induction by chronic ethanol exposure, increasing phenytoin clearance and lowering plasma concentrations. Therapeutic drug monitoring is particularly important during withdrawal or early abstinence.	[143]

**Table 8:** Alcohol-associated liver disease and cirrhosis as modifiers of hepatic transporters, protein binding and functional drug clearance.

Interacting Agents	Design, Sample, and Evidence Category	Main Finding	Clinical Interpretation	Study or Report
Alcoholic cirrhosis and hepatic drug transporters	Targeted quantitative proteomics of human liver tissue from patients with alcohol-associated cirrhosis (*n* = 27), HCV cirrhosis (*n* = 30) and non-cirrhotic control liver tissue (*n* = 36); supporting biological evidence	In alcoholic cirrhosis, hepatobiliary transporter expression changed in a transporter-dependent manner: OATP1B1 and OATP1B3 were reduced, MRP3 was increased, and several transporters showed no major change.	Alcohol-associated cirrhosis may alter transporter-mediated hepatic drug uptake and efflux, contributing to unpredictable drug exposure beyond CYP-mediated metabolism.	[38]
Chronic alcohol ingestion/alcoholic liver disease with highly bound drugs	Human plasma protein-binding study; supporting biological evidence	Binding of salicylate, sulfadiazine and phenylbutazone was normal in chronic alcohol users without overt liver disease but decreased in alcohol-induced liver disease; in alcoholic hepatitis, reduced binding correlated with serum bilirubin and albumin concentrations.	Reduced binding mainly reflects liver disease, not alcohol exposure alone.	[130]
Alcoholic cirrhosis with valproate	Clinical pharmacokinetic study (*n* = 7 with alcoholic cirrhosis); controlled human evidence	At a valproate concentration of 80 μg/mL, protein binding decreased from 88.7 ± 5.2% in controls to 70.7 ± 11.3% in patients with alcoholic cirrhosis.	Alcoholic cirrhosis may substantially increase the unbound valproate fraction, making total plasma concentrations an unreliable indicator of pharmacologically active exposure.	[131]
Liver cirrhosis with highly protein-bound CYP probe substrates	Basel phenotyping cocktail study (*n* = 36 patients with liver cirrhosis; *n* = 12 controls); controlled human evidence	Total and free plasma concentrations were compared for six CYP probe substrates. For highly protein-bound substrates (>99%; efavirenz and flurbiprofen), correlations between total- and free-concentration metabolic ratios were weak, whereas correlations were better for drugs with lower or intermediate protein binding. In cirrhosis, total plasma concentrations poorly reflected free drug concentrations for highly protein-bound drugs.	This supports the concept that liver disease can increase the pharmacologically active free drug fraction, even when total drug levels appear acceptable.	[35]
Liver cirrhosis with Basel CYP phenotyping cocktail	Clinical phenotyping study (*n* = 36 patients with cirrhosis; *n* = 12 matched controls); controlled human evidence	In Child C cirrhosis, CYP activity markers decreased for CYP1A2, CYP2B6, CYP2C19, CYP2D6 and CYP3A, whereas CYP2C9 activity was not significantly altered.	Advanced liver disease produces non-uniform impairment of CYP activity; cirrhosis may therefore make drug clearance pathway-dependent and less predictable.	[36]

**Table 9:** Alcohol use as a context-dependent modifier of drug-induced liver injury.

Interacting Agents	Design, Sample, and Evidence Category	Main Finding	Clinical Interpretation	Study or Report
Paracetamol/acetaminophen: timing-dependent hepatotoxicity				
Acetaminophen (1 g four times daily for 2 days) in newly abstinent patients with alcohol dependence	Randomized double-blind placebo-controlled trial (*n* = 201 newly abstinent alcohol-dependent patients); controlled human evidence	Acetaminophen did not increase liver injury. Day-4 AST/ALT were 38.0 ± 26.7/40.1 ± 30.9 U/L with acetaminophen versus 37.5 ± 27.6/41.9 ± 33.9 U/L with placebo; INR was similar: 0.96 ± 0.09 versus 0.98 ± 0.11.	Short-course therapeutic-dose acetaminophen did not worsen hepatic injury markers in controlled early abstinence.	[145]
Acetaminophen (4 g/day for 3 consecutive days) in newly abstinent patients with alcohol dependency	Multicenter double-blind randomized trial (*n* = 443 enrolled; acetaminophen *n* = 308, placebo *n* = 135); controlled human evidence	Acetaminophen did not worsen hepatic tests versus placebo. Peak ALT was similar with acetaminophen and placebo: 57 ± 45 versus 55 ± 48 IU/L. The study had 95% power to detect a 15 IU/L difference in ALT.	Therapeutic-dose acetaminophen did not increase short-term hepatic risk in controlled newly abstinent patients.	[24]
Sustained-release acetaminophen (1300 mg every 8 h for 11 doses) with recent alcohol abstinence	Randomized triple-blind parallel-group trial (*n* = 52 randomized; 40 completed ≥4 days; recently abstinent alcohol-dependent patients); controlled human evidence	Sustained-release acetaminophen did not significantly increase AST, ALT, or INR over 5 days. Alpha-glutathione S-transferase was lower in the acetaminophen group on day 2 by 32% and on day 3 by 29%, with differences resolving by day 4.	Sustained-release therapeutic-dose acetaminophen did not worsen hepatic injury markers in recently abstinent patients under controlled conditions.	[146]
Paracetamol with regular alcohol use	Retrospective case series (*n* = 67 regular alcohol users; paracetamol taken with therapeutic intent, reported daily doses <4 g to >15 g for 1–30 days); case-report evidence	Hepatic injury occurred after paracetamol taken with therapeutic intent; reported daily doses ranged from <4 g to >15 g for 1–30 days. Mortality was 18%, and AST exceeded 3000 U/L in 90% of cases.	Regular alcohol use may increase susceptibility to severe paracetamol hepatotoxicity, particularly with prolonged or supratherapeutic “therapeutic-intent” use.	[148]
Paracetamol shortly after alcohol cessation	Case report/short case series (*n* = 2 regular drinkers); case-report evidence	Two regular alcohol users developed acute liver failure 3–5 days after hospitalization and alcohol cessation while receiving paracetamol 4 g/day. In one patient, the measured serum paracetamol concentration was not in the toxic range.	These cases suggest that severe paracetamol hepatotoxicity may occur shortly after alcohol cessation in susceptible patients, even at therapeutic dosing.	[23]
Paracetamol (4 g/day) with ongoing heavy alcohol use	Case report (*n* = 1); case-report evidence	A 52-year-old woman with alcohol dependence developed recurrent acute liver injury while taking paracetamol and drinking heavily; AST peaked at 8015 U/L during the first episode and reached 3322 U/L during recurrence.	This case suggests caution with repeated paracetamol use in heavy drinkers, particularly when liver tests are already abnormal.	[151]
Other drug-induced liver injury contexts				
Alcohol consumption, variably defined, with anti-tuberculosis drugs	Systematic review and meta-analysis of 53 studies; observational clinical evidence	Alcohol consumption was associated with higher risk of anti-tuberculosis drug-induced liver injury (OR 1.55; 95% CI 1.19–2.04), likely by increasing hepatic oxidative/metabolic stress and reducing liver reserve.	Alcohol may increase susceptibility to anti-tuberculosis drug-induced liver injury.	[149]
Baseline alcohol use during standard anti-tuberculosis therapy	Retrospective two-center cohort study in patients starting first-line anti-TB therapy (*n* = 2624); observational clinical evidence	Alcohol use was one of six baseline predictors of anti-TB drug-induced liver injury, together with age ≥ 60 years, BMI < 18.5 kg/m^2^, extrapulmonary TB, albumin < 35 g/L, and hemoglobin < 110 g/L. The prediction model showed AUCs of 0.80, 0.75, and 0.77 in training, internal validation, and external validation cohorts.	Baseline alcohol use may increase vulnerability to anti-tuberculosis drug-induced liver injury and supports closer monitoring of liver function during treatment.	[28]
Alcohol intake >21 units/week during methotrexate therapy	CPRD cohort (*n* = 11,839 patients with rheumatoid arthritis starting methotrexate); observational clinical evidence	Alcohol intake >21 units/week was associated with transaminitis >3× ULN, adjusted HR 1.85; 95% CI 1.17–2.93, whereas ≤14 units/week was not associated with increased risk.	Higher alcohol intake may dose-dependently increase hepatotoxic risk during chronic methotrexate therapy.	[150]
Heavy alcohol consumption in idiosyncratic DILI	Prospective DILIN cohort analysis (*n* = 1198 definite, highly likely, or probable DILI cases); observational clinical evidence	Heavy drinkers had higher peak ALT and bilirubin than non-drinkers, but liver-related death or transplantation did not differ significantly.	Heavy alcohol use should not be assumed to worsen all idiosyncratic DILI outcomes; alcohol appears to be a context- and drug-specific risk modifier rather than a universal severity determinant.	[152]

## Data Availability

No new data were created or analyzed in this study. Data sharing is not applicable to this article.

## Abbreviations

ACE	Angiotensin-converting enzyme
ADH	Alcohol dehydrogenase
AKI	Acute kidney injury
ALD	Alcohol-associated liver disease
ALT	Alanine aminotransferase
ALDH	Aldehyde dehydrogenase
APAP	Acetaminophen/paracetamol
ARB	Angiotensin receptor blocker
AST	Aspartate aminotransferase
AUC	Area under the concentration–time curve
AUD	Alcohol use disorder
BAC	Blood alcohol concentration
CES1	Carboxylesterase 1
CGMS	Continuous glucose monitoring system
C_max_	Maximum plasma concentration
CNS	Central nervous system
CYP	Cytochrome P450
DILI	Drug-induced liver injury
DILIN	Drug-Induced Liver Injury Network
DMET	Drug-metabolizing enzymes and transporters
ER	Extended-release
GABA_A	Gamma-aminobutyric acid type A
GIB	Gastrointestinal bleeding
INR	International normalized ratio
LADME	Liberation, absorption, distribution, metabolism and excretion
MALA	Metformin-associated lactic acidosis
MRP3	Multidrug resistance-associated protein 3
NAPQI	*N*-acetyl-p-benzoquinone imine
NAT2	*N*-acetyltransferase 2
NMDA	*N*-methyl-D-aspartate
NMTT	*N*-methylthiotetrazole
NSAID	Nonsteroidal anti-inflammatory drug
OATP1B1	Organic anion transporting polypeptide 1B1
OATP1B3	Organic anion transporting polypeptide 1B3
OR	Odds ratio
P-gp	P-glycoprotein
PK/PD	Pharmacokinetic/pharmacodynamic
POSAMINO	Potentially serious alcohol–medication interactions in older adults
RAS	Renin–angiotensin system
ROS	Reactive oxygen species
RR	Relative risk
SGLT2	Sodium-glucose cotransporter 2
Tmax	Time to maximum plasma concentration
UGIB	Upper gastrointestinal bleeding
UGT	Uridine 5′-diphospho-glucuronosyltransferase
ULN	Upper limit of normal
